# A universal route to efficient non-linear response via Thomson scattering in linear solids

**DOI:** 10.1093/nsr/nwad136

**Published:** 2023-05-10

**Authors:** Yongzheng Wen, Flavio Giorgianni, Igor Ilyakov, Baogang Quan, Sergey Kovalev, Chen Wang, Carlo Vicario, Jan-Christoph Deinert, Xiaoyu Xiong, Joe Bailey, Min Chen, Alexey Ponomaryov, Nilesh Awari, Andrea Rovere, Jingbo Sun, Roberto Morandotti, Luca Razzari, Gabriel Aeppli, Junjie Li, Ji Zhou

**Affiliations:** State Key Laboratory of New Ceramics and Fine Processing, School of Materials Science and Engineering, Tsinghua University, Beijing 100084, China; Paul Scherrer Institut, Villigen PSI 5232, Switzerland; Helmholtz-Zentrum Dresden-Rossendorf, Dresden 01328, Germany; Beijing National Laboratory for Condensed Matter Physics, Institute of Physics, Chinese Academy of Sciences, Beijing 100190, China; Helmholtz-Zentrum Dresden-Rossendorf, Dresden 01328, Germany; State Key Laboratory of New Ceramics and Fine Processing, School of Materials Science and Engineering, Tsinghua University, Beijing 100084, China; Paul Scherrer Institut, Villigen PSI 5232, Switzerland; Helmholtz-Zentrum Dresden-Rossendorf, Dresden 01328, Germany; State Key Laboratory of New Ceramics and Fine Processing, School of Materials Science and Engineering, Tsinghua University, Beijing 100084, China; Paul Scherrer Institut, Villigen PSI 5232, Switzerland; Institut de Physique, École Polytechnique Fédérale de Lausanne (EPFL), Lausanne CH-1015, Switzerland; Helmholtz-Zentrum Dresden-Rossendorf, Dresden 01328, Germany; Helmholtz-Zentrum Dresden-Rossendorf, Dresden 01328, Germany; Helmholtz-Zentrum Dresden-Rossendorf, Dresden 01328, Germany; Institut National de la Recherche Scientifique (INRS), Centre Énergie, Matériaux et Télécommunications (EMT), Varennes J3X1P7, Canada; State Key Laboratory of New Ceramics and Fine Processing, School of Materials Science and Engineering, Tsinghua University, Beijing 100084, China; Institut National de la Recherche Scientifique (INRS), Centre Énergie, Matériaux et Télécommunications (EMT), Varennes J3X1P7, Canada; Institut National de la Recherche Scientifique (INRS), Centre Énergie, Matériaux et Télécommunications (EMT), Varennes J3X1P7, Canada; Paul Scherrer Institut, Villigen PSI 5232, Switzerland; Institut de Physique, École Polytechnique Fédérale de Lausanne (EPFL), Lausanne CH-1015, Switzerland; Department of Physics and Quantum Center, ETH Zürich, Zürich CH-8093, Switzerland; Beijing National Laboratory for Condensed Matter Physics, Institute of Physics, Chinese Academy of Sciences, Beijing 100190, China; State Key Laboratory of New Ceramics and Fine Processing, School of Materials Science and Engineering, Tsinghua University, Beijing 100084, China

**Keywords:** non-linear optical materials, terahertz harmonic generation, Thomson scattering, magneto-electric coupling, metamaterial

## Abstract

Non-linear materials are cornerstones of modern optics and electronics. Strong dependence on the intrinsic properties of particular materials, however, inhibits the at-will extension of demanding non-linear effects, especially those second-order ones, to widely adopted centrosymmetric materials (for example, silicon) and technologically important burgeoning spectral domains (for example, terahertz frequencies). Here we introduce a universal route to efficient non-linear responses enabled by exciting non-linear Thomson scattering, a fundamental process in electrodynamics that was known to occur only in relativistic electrons in metamaterial composed of linear materials. Such a mechanism modulates the trajectory of charges, either intrinsically or extrinsically provided in solids, at twice the driving frequency, allowing second-harmonic generation at terahertz frequencies on crystalline silicon with extremely large non-linear susceptibility in our proof-of-concept experiments. By offering a substantially material- and frequency-independent platform, our approach opens new possibilities in the fields of on-demand non-linear optics, terahertz sources, strong field light–solid interactions and integrated photonic circuits.

## Teaser

Metamaterials featuring locally enhanced non-linear Thomson scattering offer a practical and universal method for efficiently stimulating non-linear responses in linear solid materials.

## INTRODUCTION

Optical non-linearities are among the most fundamental manifestations of strong light–matter interactions, lying at the core of photonics and optical information technology [1,2]. In particular, the underlying second-order optical non-linear processes, such as optical parametric oscillation and second-harmonic generation (SHG), have been extensively studied across wide material and spectral ranges, and have led to flourishing applications in both the classical and quantum regimes, including optical-frequency converters, compact quantum sources, non-linear spectroscopy and sensing [1,3–6]. These non-linear phenomena usually arise from intrinsic properties of specific materials, which are often not compatible with desirable platforms such as silicon technology, and not engineered to respond at arbitrary frequencies. In particular, terahertz (THz) frequencies, ideally bridging electronics and optics, seem to be a missing piece in the well-established field of non-linear optics. Their rich potentials are extremely desired from both fundamental and applied perspectives, for example, ultrahigh speed electronics in picosecond response, integrated tunable THz sources, and THz dynamics in topological polar materials [7–9], benefiting various applications such as wireless communication, data processing and storage, and radar-based systems, among others [10–12]. The canonical demand of symmetry breaking for second-order non-linearity rules out most naturally occurring candidates, including advanced platforms such as two-dimensional materials and Dirac semimetals, where THz odd-order harmonic generation has recently been demonstrated [13–15]. Exciting new work at a cryogenic temperature suggests that superconducting films can realize THz SHG [16] while suffering from a very limited operating frequency band, as some very early pioneering studies show [17,18]. Despite substantial efforts, the goal of a practical (most notably silicon-compatible) approach for THz second-order non-linear responses is yet to be achieved due to the lack of suitable non-linear materials.

In the optical regime, there is a long tradition of using plasmonic nanostructures to excite and control non-linear effects by locally enhancing the electric field of the optical radiation [19–23]. While these magnetic and electric resonances clearly advance non-linear optics [24,25], the unavoidable requirement for the host media to possess non-linear responses severely limits their extensions in both spectral and material domains. Non-linear Thomson scattering, on the other hand, is one of the most fundamental mechanisms in electrodynamics, which emits radiation at harmonics of the exciting light frequency due to the non-linear quiver of electrons under the strong effect of the magnetic field. After decades of theoretical studies, it has recently become possible to experimentally realize non-linear Thomson scattering with relativistic electrons in vacuum and an ultrahigh-peak-power laser [26,27]. Although theoretical studies have predicted that it applies to the whole electromagnetic spectrum and may not be prohibited for symmetry reasons [28], its intrinsic weakness makes it play virtually no role in natural solids because of the slow motion of electrons and weak magnetic contribution to the Lorentz force. Artificial metamaterials can be used to locally redistribute and enhance both magnetic and electric fields. This, in turn, may open up a new avenue for generating very efficient non-linear responses by exciting non-linear Thomson scattering in solids [29,30], which is an essential but long-term challenge in electrodynamics and exceptionally beneficial in THz and semiconductor technologies. Here we introduce a metamaterial-based approach to enabling efficient non-linear response, which neither depends on the material non-linear properties nor on the frequency range. Via the coupling of a metamaterial resonator and a semiconductor, impact ionization, a well-known carrier-multiplication process, is stimulated by optical irradiation. The locally enhanced magnetic field then interacts with the charged carriers and induces strong non-linear Thomson scattering, exciting efficient second-order non-linear responses in symmetric linear solids. We verify this approach by experimentally achieving SHG at THz frequencies and on silicon substrate, which are both extremely challenging in the conventional context of optical non-linear materials. The strength of non-linear Thomson scattering in a solid is orders of magnitude higher than that in free electrons in a vacuum, and it results in an extremely large THz second-order non-linear susceptibility at room temperature, exceeding that of most typical asymmetric non-linear media. Besides impact ionization, we demonstrate that other methods of free electron injection, such as the commonly used impurity doping techniques, can generate efficient non-linear responses as well.

## RESULTS

**Figure 1.**
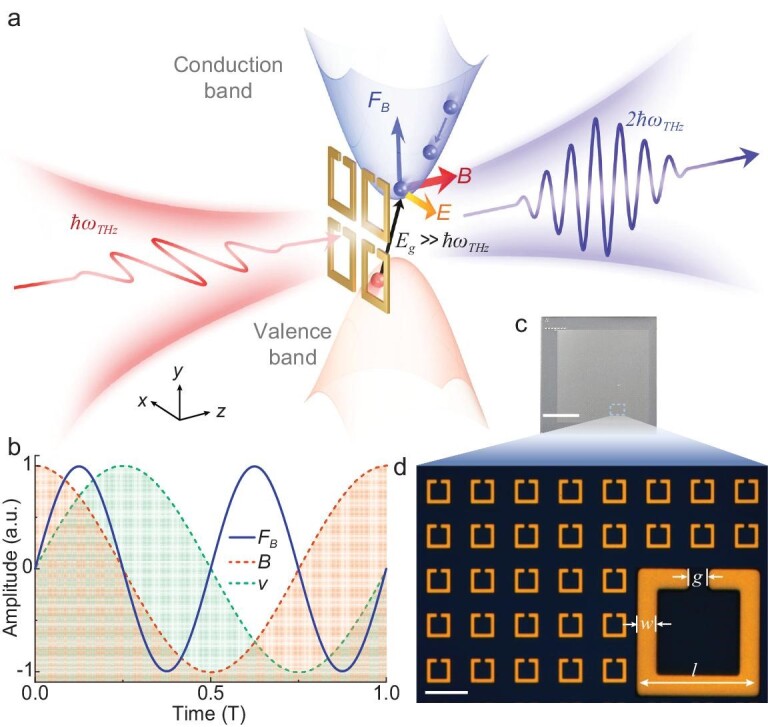
Principle and design of metamaterial-based optical non-linearity. (a) Schematic of the physical processes behind the proposed non-linear mechanism, and orientations of the local electric field, *E*, and magnetic field, *B*, as well as of the Lorentz force, *F_B_*. (b) Relationship between the Lorentz force, the magnetic field and drift velocity, *v*, in time domain. The time is normalized to one period T of fundamental oscillation. (c and d) Photograph (c) and microscopic image (d) of the metamaterial operating at 0.68 THz with a lattice constant of 53 μm (scale bars: (c) 50 mm; (d) 50 μm), where the inset shows a single unit cell: *l* = 27.5 μm, *w* = 4 μm, *g* = 5 μm.

Figure 1a illustrates the principles of metamaterial-based optical non-linearity. For a strong electric field with frequency *ω* applied to a material with a bandgap, including semiconductors and insulators, the electrons in the conduction band gain a large ponderomotive energy, which despite the very low photon energy (for example, THz photons in millielectronvolts energy) may dramatically exceed the bandgap, thus stimulating impact ionization. As a result, bound electrons are scattered out of the valence band into the conduction band and produce an avalanche of new electron-hole pairs, featuring velocity, ***v***, oscillating at frequency *ω*[31,32]. The strong magnetic field, ***B***, oscillating at the same frequency *ω*, will then lead to strong non-linear Thomson scattering based on a magnetic contribution to Lorentz force, ***F_B_*** = *q****v*** × ***B*** at frequency 2*ω* (*q* is the elementary charge), which entails evident non-linear responses, such as SHG. Due to the much lower mobility of holes, only free electrons are considered. Figure 1b illustrates the relation between the drift velocity, magnetic field and Lorentz force. Although this non-linear process is irrelevant for common solids due to the unrealistically high field required [31,33], the localized enhancement of the electric and magnetic fields in a metamaterial relaxes the applied field strength requirement and allows the process to take place (see Supplementary Note 1). As the two dominant processes of impact ionization and non-linear Thomson scattering have very few requirements on specific material composition or frequency band, this non-linear mechanism can be generalized to a very broad range of materials, and offers unique possibilities to directly tailor its properties by judicious engineering of the chemical composition and structural morphology of the metamaterial.

**Figure 2.**
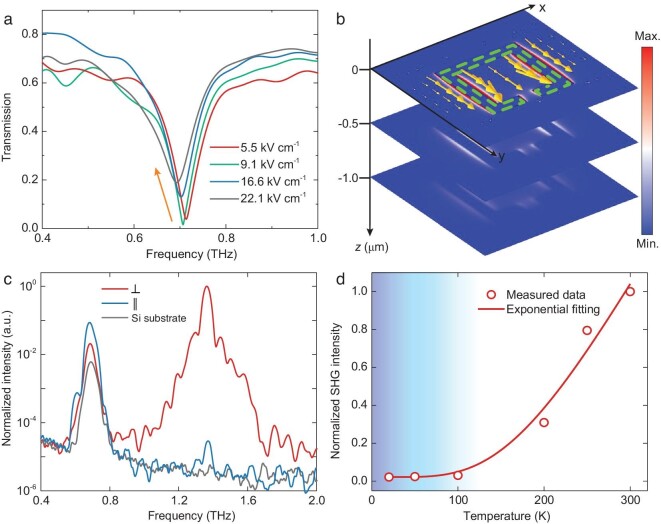
Experimental results of the metamaterial induced SHG. (a) Measured transmission spectra of the metamaterial under different strengths of THz pump. The arrow indicates direction of increasing THz field strength. (b) Simulated distribution of the Lorentz force in the metamaterial at 0.68 THz with a normalized scale. Here, the arrows mark the direction of the force, and the shape of the SRR is represented by the green dashed line. (c) Cross-polarized spectra emitted by the metamaterial when the pump pulses are polarized orthogonal (along the *x* axis, red curve) and parallel (along the *y* axis, blue curve) to the gap of the SRR, as well as for the bare silicon substrate alone (black curve). All the spectra are recorded at room temperature. (d) Dependence of the SHG intensity on the sample temperature with the maximum pump of the fundamental THz frequency, ranging from 20 K to 300 K with an exponential fitting curve. The intensity is normalized to the peak intensity of the SHG measured at room temperature.

Guided by the theoretical model, we designed and fabricated a proof-of-principle metamaterial resonating at 0.68 THz (Fig. 1c and d). We utilize gold and high-purity single crystalline (001) silicon to form the split-ring resonator (SRR) array and substrate, respectively. The symmetric crystal structure of both materials eliminates the possibility of second-order non-linearity directly arising from the bulk constituents. The transmission spectra of the metamaterial were first characterized with table-top terahertz spectroscopy. At the lowest field, the impact ionization is not significant, and there is a characteristic dip of the SRR at the resonance (Fig. 2a). As the incident field increases the resonance frequency redshifts, and the transmission increases and broadens. These results suggest a large increase in silicon conductivity beneath the SRR (see Supplementary Note 2), indicating an evident population of free electrons via impact ionization [32,34,35]. The metamaterial samples were then excited by an electron accelerator-based linearly polarized source (TELBE) [36] with a quasi-monochromatic spectrum centered at 0.68 THz and a maximum peak field of 98.9 kV cm^−1^ (see Materials and Methods, and Supplementary Fig. 1). The transmitted and emitted THz signals are probed through electro-optical sampling [37]. Resonating at the pump THz frequency, the SRR significantly enhances both the electric and magnetic fields (Supplementary Fig. 3). Given an average field enhancement factor of 15, the local electric field reaches an amplitude of 1.48 MV cm^−1^ and provides a ponderomotive energy of 202.6 eV, which is 180.9 times larger than the bandgap of silicon (*E_g_* = 1.12 eV) and leads to avalanche multiplication of the free electrons with high velocity (see Supplementary Note 1). Induced by the circulating currents of the SRR, the out-of-plane oscillatory magnetic field further acts on the newly generated electrons, producing a significant second-order Lorentz force. Based on the distributions of the Lorentz force, we estimated that the non-linear Thomson scattering is largely confined to the 0.5 μm thickness in the vicinity of the substrate–SRR interface (Fig. 2b, see Supplementary Note 3). The force drives the free electrons to oscillate at a frequency 2*ω*, resulting in SHG that is cross-polarized with respect to the fundamental wave due to the cross-product nature of the Lorentz force.

Figure 2c shows the cross-polarization spectra of the sample at room temperature. With the incident polarization orthogonal to the gap of the SRR (along the *x* axis), the SHG at 1.36 THz is clearly observable with an amplitude conversion efficiency of 3.5 × 10^−4^ (Supplementary Fig. 4). The degree of linear polarization of the emitted SHG is found to be in the range of 92.3%–99.0% with respect to the fundamental polarization (Supplementary Fig. 5), confirming the theoretical prediction of a cross-polarized SHG. As expected, when rotating the pump polarization parallel to the gap of the SRR (along the *y* axis), the SHG disappears with the vanishing resonance (Fig. 2c, blue curve). No second-harmonic signal is visible in the spectrum of the bare substrate (Fig. 2c, black curve) due to the very few free electrons and the absence of non-linear Thomson scattering in the high-purity silicon. Because of the multiple reflections in the substrate, the SHG shows a sharp peak with full width at half maximum (FWHM) of 0.033 THz, narrower in this sense than the fundamental pump, 0.065 THz. The broad bandwidth of the tail regions (whose visibility is enhanced because of the logarithmic scale of Fig. 2c) derives from the short time duration of the initial SHG pulse, which results from folding the incident beam profile with the impact ionization threshold (see Supplementary Fig. 4). The transmitted fundamental signals are significantly suppressed by the cross-polarized detection and the bandpass filters in the experimental set-up (see Materials and Methods), leading to a weaker intensity than the SHG shown in the spectra. To better understand the role of free electrons in the non-linear process, we measured the second-harmonic amplitude at different temperatures, to alter the density of the intrinsic conduction-band carriers in silicon (Fig. 2d). When cooling the sample, significantly fewer intrinsic electronic carriers are thermally excited in the conduction band, and the number of newly generated electrons also decreases dramatically. The SHG intensity thus decreases with temperature as well, following an exponentially activated form, phenomenologically accounting for the combined temperature dependence of various ingredients including not only the impact ionization rate and mobility, but also the metamaterial resonance [38,39] (see Supplementary Note 5). It demonstrates that the non-linear responses result from the light–electron interactions as indicated in the Thomson scattering mechanism. All of these results definitively support the assumption that SHG originates from the joint action of impact ionization and non-linear Thomson scattering, both enhanced by the metamaterial proposed here. The strongly non-relativistic motion of the electrons in solids breaks the consensus that non-linear Thomson scattering can only occur in electrons featuring near-light speed.

**Figure 3.**
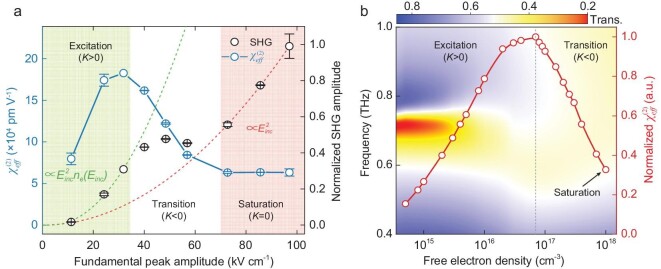
Dependence of THz SHG on the peak amplitude of the fundamental pump field. (a) Measured dependence of the THz SHG amplitude and $\chi _{\textit{eff}}^{(2)}$ on the fundamental pump amplitude. The dashed lines mark the theoretical expectation for the THz SHG amplitude in the excitation (green) and saturation (red) regions. Error bars indicate standard deviations from multiple measurements. (b) Simulated transmission spectra (contour map) and $\chi _{\textit{eff}}^{(2)}$ (red line) of the metamaterial at different free electron densities induced by impact ionization in the silicon substrate ($\chi _{\textit{eff}}^{(2)}$ is normalized to its maximum).

The combination of impact ionization and non-linear Thomson scattering in solids yields a unique amplitude dependence of the SHG on the pump field, *E_inc_* (Fig. 3a), which has not been reported in the conventional gaseous media according to our knowledge. Considering that the magneto-electric coupling is constrained to a 0.5 μm thick layer, it is possible to extract the effective second-order susceptibility, $\chi _{\textit{eff}}^{(2)}$, at different pump fields (see Supplementary Note 6). Its slope $K = \frac{{d\chi _{\textit{eff}}^{(2)}}}{{d{E}_{\textit{inc}}}}$ is defined so as to clarify the SHG features as well as the underlying physics. There are three evident regions in the SHG evolution versus pump amplitude that could be associated with the sign of the slope, which we name excitation, transition and saturation. In the excitation region with the pump field below 32.5 kV cm^−1^, a positive *K* (*K* > 0) is obtained. In this limit, new free electrons start to be excited by impact ionization, but the total number of carriers at different pump fields, *n_e_(E_inc_)*, is low enough to not significantly affect both the local field enhancement of the SRR and the carrier mobility in silicon (Fig. 3b). Together with the second-order behavior, the electric field of the THz SHG, *E_2__ω_*, follows the relation ${E}_{2\omega } \propto E_{\textit{inc}}^2{n}_e\!({E}_{\textit{inc}})$ (Fig. 3a, green curve), as presented in our theoretical model (see Supplementary Note 6). The non-linear susceptibility increases with the intensity of the incident fundamental wave and reaches 1.9 × 10^5^ pm V^−1^. In the transition region, for increasing pump fields, the sign of *K* becomes negative (*K* < 0). Here, the density of the populated electrons is high enough to degrade both the resonance strength and the carrier mobility. Balanced by the stronger pump, the SHG amplitude presents a plateau-like response with a small variation in strength and a decay of $\chi _{\textit{eff}}^{(2)}$. In the saturation region, with a pump field larger than 74.2 kV cm^−1^, no more electrons are further produced as the impact ionization is saturated, and the substrate behaves similarly to n-doped silicon with a constant doping concentration. Solely dominated by non-linear Thomson scattering, the SHG grows quadratically with an increase in illumination (Fig. 3a, red curve), which is characteristic of standard perturbative second-order non-linearity. Here, a constant $\chi _{\textit{eff}}^{(2)}$ of 6.5 × 10^4^ pm V^−1^ is extracted in the zero-slope region (*K* = 0). We also investigated the bandwidths of the SHG at different pump fields, and they all show similar behavior. In particular, the broadband tail regions shown in Fig. 2c are found to be always present, which demonstrates that the process of impact ionization occurs even at the weakest pump in our measurements.

We numerically modeled the resonant behavior and $\chi _{\textit{eff}}^{(2)}$ of the metamaterial as shown in Fig. 3b (see Materials and Methods, and Supplementary Note 7). With more and more electrons produced by increasing the driving THz field, the same unique evolution of the non-linear susceptibility observed in the measurements is numerically reproduced. At saturation, the constant non-linear susceptibility is calculated to be roughly one third of the maximum value, in excellent agreement with the experimental results.

We note that the non-linear Thomson scattering in solid-state metamaterials is surprisingly strong. Conventionally, the physical nature of Thomson scattering depends on the incident electromagnetic field amplitude, which becomes evident when the normalized vector potential *a_0_* is of the order of unity. At the incident electric field of *E_inc_* = 32.5 kV cm^−1^, where the largest effective susceptibility is achieved, we obtain an effective value of *a_0_* for the solid-state metamaterials as 0.23, and the *a_0_* value for the rest electron in vacuum is only 4.5 × 10^−4^ (see Supplementary Note 8). This suggests that the implementation of metamaterial fundamentally relaxes the field strength requirements for non-linear Thomson scattering to take place, equivalent to applying over-five-orders-of-magnitude-stronger terahertz radiation, which is currently unrealistic. A practical femtosecond laser at a wavelength of 800 nm in a power level of 10^17^ W cm^−2^ is expected to reach a similar *a_0_* strength of 0.26 [26,27], which is over 10 orders of magnitude more intense than the source we used and exceeds the damage thresholds of materials. The charged carriers in condensed matters are usually orders of magnitude denser than those in gaseous and plasma ones, further boosting non-linear Thomson scattering.

Extremely strong Thomson scattering leads to very large effective second-order susceptibility, exceeding any other reported room-temperature values at THz frequencies, to the best of our knowledge. The measured second-order susceptibility of the sample is four and two orders of magnitude higher than those of GaAs and LaTiO_3_ respectively [17]. Moreover, the efficiency of the non-linear responses of the metamaterial can surely be further improved. Superconductors present a second-order non-linear susceptibility as large as 1.27 × 10^7^ pm/V at a cryogenic condition [16], almost two orders of magnitude higher than our room-temperature results. One possible strategy would be increasing the effective thickness by fabricating a stack of alternating resonators and semiconductor layers. The advanced deep-learning-based photonic design may help to propose a metamaterial structure with better performance as well [40]. On the other hand, the damage threshold of the composite materials and possible high-order harmonic generation may constrain the absolute conversion efficiency of the SHG at very intense pump, resembling the saturation of the conventional non-linear materials.

SRR-like structures have been employed to generate SHG at near-infrared frequencies [41,42], with a series of theoretical and experimental studies demonstrating that the principal origin is the surface non-linearity of noble metals [43,44]. However, in our case at THz frequencies, the contribution of the surface non-linearity is negligible, as it only happens in one or two atomic layers at the physical interface [45,46], which is less than 1 nm thick and far thinner than the 500 nm thick interaction layer of bulk non-linear Thomson scattering. The non-linear conversion efficiency from non-linear Thomson scattering is estimated to be over seven orders of magnitude higher than that from the gold surface in the THz metamaterial (see Supplementary Note 9). The unique amplitude dependence also differentiates our mechanism from others, including the surface non-linearity, in which a standard quadratic dependence is usually expected [41,47].

**Figure 4.**
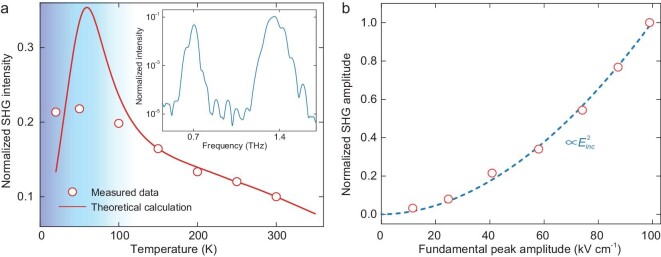
SHG from the doped-silicon metamaterial. (a) Dependence of the measured SHG intensity (red dots) on the sample temperature ranging from 20 K to 300 K, with the maximum pump of the fundamental THz frequency. The red curve marks the theoretical calculation, and the inset is the cross-polarized spectrum emitted by the doped-silicon metamaterial at room temperature. The intensity is normalized to the peak intensity of the SHG from the undoped-silicon sample measured at room temperature. (b) Dependence of THz SHG amplitude on fundamental pump amplitude at room temperature: measured values (red dots) and the quadratic fitting (blue dashed curve). SHG amplitude is normalized to its maximum.

According to the principle of non-linear Thomson scattering, other methods of free electron injection are also expected to generate a second-harmonic signal besides impact ionization, such as the commonly used impurity doping techniques. By implanting phosphorus ions on the high purity silicon substrate, we prepared the same SRR structure on a continuous n-doped film (see Materials and Methods), where the free electrons, interacting with the magnetic field, come from the thermal ionization of impurities instead of the impact ionization. Thus, the features of the non-linear responses in the doped-silicon sample are evidently distinct from those in the undoped one. As no threshold is involved, the second-harmonic peak from the doped-silicon sample at room temperature shows an FWHM of 0.092 THz (inset of Fig. 4a), meeting the conventional expectation of $\sqrt 2 $ times wider than that of the fundamental pump. Its amplitude conversion efficiency is 1.58 × 10^−4^ (see Supplementary Fig. 8), leading to almost one order of magnitude lower intensity than that of the undoped-silicon sample because of the high THz loss in the doped layer. When cooling the doped-silicon sample, the SHG intensity increases (Fig. 4a), which is the opposite of what happens to the undoped-silicon one. This is because the electron mobility increases but the density of the free electrons originating from the impurities varies little at temperatures above 100 K, where the theoretical calculations are perfectly consistent with the measured results. At the cryogenic region below 100 K, although some cryogenic effects were not fully considered, our room-temperature model still properly captures the key experimental features, for example, the SHG intensity reaches its maximum at ∼50 K and then drops with a decrease in temperature (see Supplementary Note 10). As the thermally ionized free electrons in the doped silicon are independent of the terahertz electric field, and non-linear Thomson scattering solely dominates, the SHG amplitude dependence on the pump field follows the iconic quadratic law of the second-order response (Fig. 4b).

The underlying differences in temporal coherence, and temperature and power dependences between the doped and undoped silicon samples indicate that the well-established strategies to tailor electronics and photonics can be compatibly applied to modulate solid-state non-linear Thomson scattering with ultrahigh freedoms, which are highly desired but very challenging in relativistic media like plasma. This advantage potentially manifests many advanced functions, such as active modulation and field programmability.

A set of measurements was further performed to demonstrate that metamaterial-enabled non-linear Thomson scattering is independent of chemical compositions and applicable to different frequency bands. By replacing the substrate with c-cut sapphire and maintaining the same geometry for the gold metamaterial, we obtained a weak but detectable THz SHG, which demonstrates that our mechanism can be extended to other semiconductors and insulators (see Supplementary Fig. 9). The fact that we could demonstrate SHG from single crystalline silicon and sapphire, well-known inversion symmetric compounds, shows how even-order non-linear effects are possible for materials featuring inversion symmetry, by exploiting the magneto-electric mechanism introduced herein. The much-lower conversion efficiency of the sapphire-based sample experimentally rules out the possibility that second-order non-linear responses principally arise from the surface non-linearity of gold SRRs as well. If surface symmetry breaking of gold dominated, one would expect similar SHG strength due to the same SRR structure. High-order polarizations in centrosymmetric materials, such as electric quadrupole effects, may also support second-order optical responses [28], but their weakness and dependence on specific optical geometry often make them challenging to excite and detect [48,49]. Our silicon-based structures, which are pumped by a normally incident THz wave, largely exclude the likelihood of such effects as well.

The proof-of-concept experiment at 0.68 THz can be conveniently extended to other THz frequencies by simply scaling the metamaterial geometry. We prepared undoped-silicon-based metamaterial samples working at the fundamental frequencies of 0.5 THz and 1.0 THz, and the corresponding SHG emissions were experimentally observed with a similar set-up (see Supplementary Figs 10 and 11). Although our demonstrations were carried out at THz frequencies, this platform can be extended, in principle, to a very broad spectral regime, from microwave to mid-infrared, because the proposed non-linear responses via non-linear Thomson scattering are theoretically expected to cover most electromagnetic wave frequencies. At infrared frequencies, one may design an all-dielectric metamaterial structure to achieve high efficiency. Dielectrics possess low loss at high frequencies and thick interaction thickness due to unique resonant modes, such as Fano resonance and quasi-bound-state-in-continuum (quasi-BIC) mode, both of which significantly benefit the conversion efficiency.

## DISCUSSION

We note that notwithstanding our demonstration with a free electron laser drive, THz SHG and solid-state non-linear Thomson scattering can also be stimulated by standard THz sources, such as the commonly used optical rectifiers driven by femtosecond lasers. Today's table-top THz sources can show comparable or even stronger peak spectral density per unit wavenumber than the narrow-band TELBE source we used [7,31,50] (see Supplementary Note 2). The SRR incorporated on a semiconductor or dielectric substrate here is only an example. Our approach allows second-order non-linearity to cover the vast majority of solids regardless of the crystalline structure, as long as they can intrinsically or extrinsically provide free carriers. Any metamaterial design satisfying the basic requirements of the magneto-electric mechanism presented above can theoretically excite non-linear Thomson scattering. Consequently, by properly structuring the geometry and choosing the compositions of the metamaterial, the resulting non-linear emission, such as phase, polarization and intensity, can be precisely manipulated and actively modulated with extremely high freedom.

In summary, we have introduced a universal route to efficient non-linear responses based on the excitations of the non-linear Thomson scattering mechanism in linear solids, which does not rely on specific material properties nor excitation frequencies. This suggests a compact, tailorable, and controllable platform for non-linear optics, and helps to fill the special gaps at the terahertz and mid-infrared frequencies. This new approach also aids in the design of myriad novel non-linear functionalities, such as the non-linear spatiotemporal vortex beam generator and non-linear elements for optical deep neural networks [51,52]. The occurrence of the non-linear response on a subwavelength scale, in common materials, together with the gate-controlled carrier populations, opens a new pathway to integrating non-linear optoelectronics with silicon-based electronics and photonics. Our approach brings non-linear Thomson scattering from high-energy physics to integrated systems with an over-ten-orders-of-magnitude reduction in pump intensity. The solid-state platform is far superior to the relativistic media for studying and modulating strong field light–matter interactions and developing novel light sources. By incorporating the metamaterial with different materials, our findings provide potential to achieve many other singular magnetic responses, including dynamic excitations and manipulations of spin waves and skyrmions [9,53], which contain rich physics but were difficult to reveal.

## MATERIALS AND METHODS

### Sample fabrication

The samples were micro-fabricated using double-sided polished silicon and sapphire substrates. The 500 μm thick high purity (001) silicon wafer featured a resistivity over 20 kΩ·cm for a high THz transmission, while the 400 μm thick sapphire wafer was oriented along the c-axis. For the samples on the undoped silicon and sapphire substrates, the SRR array structure was patterned on the substrate with standard photolithography. Subsequently, a 20/300 nm thick Ti/Au film was deposited by e-beam evaporation, followed by a lift-off process in acetone, leading to the final samples. For the samples fabricated on doped silicon, phosphorous ions were implanted in the high-purity silicon substrate with an energy of 80 keV and a dose of 4 × 10^13^ cm^−2^, followed by annealing at 1000°C in a N_2_ atmosphere for 30 min. The subsequent processes were the same as those described above, including photolithography, e-beam evaporation and lift off.

### Simulation settings

The simulation setting details are available in the Supplementary Data. In general, the metamaterial was simulated using a commercial finite-element package (COMSOL Multiphysics). The substrate materials used were high-purity silicon (dielectric constant of 11.7 and free electron density of 2 × 10^11^ cm^−3^) and sapphire (dielectric constant of 11.5 and loss tangent of 8.6 × 10^−5^), respectively. Gold conductivity was set to 4.1 × 10^7^ S/m. The n-doped silicon film was modeled with a doping concentration of 3 × 10^17^ cm^−3^ and a thickness of 300 nm. Carrier multiplication caused by the impact ionization process was considered by adding a conductive slab in the substrate. To take the magneto-electric coupling into consideration, the slab was modeled with an anisotropic conductivity tensor as follows


(1) \begin{eqnarray*} \tilde{\sigma }({B}_0) = \tilde{\sigma }(\omega )\left[ {\begin{array}{@{}*{3}{c}@{}} {\frac{1}{{1 + {\beta }^2}}}&\quad { - \frac{\beta }{{1 + {\beta }^2}}}&\quad 0\\ {\frac{\beta }{{1 + {\beta }^2}}}&\quad {\frac{1}{{1 + {\beta }^2}}}&\quad 1\\ 0&\quad 0&\quad 1 \end{array}} \right], \end{eqnarray*}

where $\beta = {\tilde{\mu }}_e\!(\omega )\!{B}_0$, *B_0_* is the local magnetic field amplitude, and $\tilde{\sigma }(\omega )$ and ${\tilde{\mu }}_e\!(\omega )$ are the conductivity and mobility at the fundamental angular frequency *ω*, respectively. The anisotropic model of the conductivity is generally applicable to most conductors and semiconductors. It does not affect the universality of our mechanism for non-linear responses.

### Experimental set-up

The room-temperature transmission spectra were measured using table-top strong-field terahertz time-domain spectroscopy based on a femtosecond laser pumping a 2-[3-(4-hydroxystyryl)-5,5-dimethylcyclohex-2-enylidene]malononitrile (OH1) crystal for generation and a ZnTe crystal for detection [50]. The relative humidity was kept below 5% by dry N_2_ purging.

The SHG experiments were conducted at ambient conditions using the TELBE THz facility. The schematic of the set-up is plotted in Supplementary Fig. 1. A set of THz bandpass filters and wire-grid polarizers were used to suppress the background SHG signal and modulate the fundamental pump. A standard electro-optical sampling (EOS) technique was employed to measure the THz field, using a 2.0 mm thick ZnTe crystal pumped by a femtosecond laser, which can only detect THz fields below 2.4 THz. Therefore, we chose a maximum fundamental frequency of 1.0 THz, resulting in a SHG frequency of 2.0 THz. We cooled the sample down using a liquid helium cryostat for low-temperature measurements with the temperature tuned from 20 K to 300 K. The experimental set-up details are available in the Supplementary Data.

## Supplementary Material

nwad136_Supplemental_File
