# Supplementary material for: A universal route to efficient non-linear response via Thomson scattering in linear solids
Source: Natl Sci Rev. 2023 May 10;10(7):nwad136. doi: 10.1093/nsr/nwad136 (PMC10313094; doi:10.1093/nsr/nwad136)
Supplement: nwad136_Supplemental_File [file nwad136_supplemental_file.pdf]

Supplementary Information

A universal route to efficient nonlinear response via Thomson scattering in linear solids

Yongzheng Wen[1], Flavio Giorgianni[2], Igor Ilyakov[3], Baogang Quan[4], Sergey Kovalev[3], Chen Wang[1], Carlo Vicario[2], Jan-Christoph Deinert[3], Xiaoyu Xiong[1], Joe Bailey[2,5], Min Chen[3], Alexey Ponomaryov[3], Nilesh Awari[3], Andrea Rovere[6], Jingbo Sun[1], Roberto Morandotti[6], Luca Razzari[6], Gabriel Aeppli[2,5,7], Junjie Li[4] and Ji Zhou[1,*]

[1]State Key Laboratory of New Ceramics and Fine Processing, School of Materials Science and Engineering, Tsinghua University, Beijing 100084, China;

[2]Paul Scherrer Institut, Villigen PSI 5232, Switzerland;

[3]Helmholtz-Zentrum Dresden-Rossendorf, Dresden 01328, Germany;

[4]Beijing National Laboratory for Condensed Matter Physics, Institute of Physics, Chinese Academy of Sciences, Beijing 100190, China;

[5]Institut de Physique, École Polytechnique Fédérale de Lausanne (EPFL), Lausanne CH-1015, Switzerland;

[6]Institut National de la Recherche Scientifique (INRS), Centre Énergie, Matériaux et Télécommunications (EMT), Varennes J3X 1P7, Canada;

[7]Department of Physics and Quantum Center, ETH Zürich, Zürich CH-8093, Switzerland

***Corresponding author.** E-mail: zhouji@tsinghua.edu.cn

Simulation Setting Details

The metamaterial was modelled and simulated by means of a commercial finite-element package (COMSOL Multiphysics). The single unit cell was simulated with periodic boundary conditions in the *x* and *y* directions, and the interfaces for transmitting and receiving the pump wave were set on the top and bottom boundaries, respectively. The linear response of the metamaterial samples was simulated in the frequency domain. Under normal illumination via a linearly polarized plane wave from the top, the transmission (*T*) spectra can be obtained from the S-parameters via the relation $T = |S_{21}|$. The polarization is assumed to be perpendicular to the gap of the SRR (along the *x* axis), and the total frequency band ranges from 0.1 THz to 1.3 THz with steps of 2.0 GHz.

The nonlinear response of the samples was simulated in the time domain via a linearly polarized Gaussian pulsed plane wave impinging from the top, with a polarization perpendicular to the gap of the SRR. The expression for the electric field is $\vec{E}_x = \vec{E}(\omega)\cos(\omega t - k_\omega z)e^{-(\frac{t-t_0}{\Delta t})^2}$, where $\vec{E}(\omega)$ is the peak amplitude of the electric field, k_ω is the vacuum wavenumber at the angular frequency ω, t is time, z is the spatial coordinate along the z axis. The parameters used to describe the Gaussian pulse, t_0=11.4 ps and Δt=6.0 ps, were extracted from the TELBE laser characterization. The maximum peak amplitude of the electric field was fixed to 98.9 kV cm^{-1}, consistent with the measured value. The angular frequency is $2\pi\times0.68\times10^{12}$ rad/s. A total time of 40 ps was simulated with steps of 10 fs.

Experimental Setup Details

The transmission spectra of the metamaterial samples were measured with a table-top strong-field terahertz time-domain spectroscopy setup[50]. A Ti: sapphire femtosecond laser system with a central wavelength of 800 nm and a repetition rate of 1 kHz is separated into two beams by a beam splitter. One beam is used to pump a commercial optical parametric amplifier to deliver a laser beam at a wavelength of 1550 nm. The linearly polarized strong-field terahertz pulse is emitted from a 2-[3-(4-hydroxystyryl)-5,5-dimethylcyclohex-2-enylidene]malononitrile (OH1) crystal pumped by the beam with the wavelength of 1550 nm. A standard electro-optical sampling (EOS) technique was employed to measure the THz field, using a 2.0 mm thick ZnTe crystal pumped by the other laser beam centered at the wavelength of 800 nm. A bandpass filter is used when

characterizing the peak amplitude of the THz wave. The energy of the THz pulse is controlled by three wire-grid THz polarizers. A 50 μm thick GaP crystal was used to characterize the full frequency-domain spectrum of the THz pulse. All the measurements were carried out at room temperature under ambient pressure, and the relative humidity was kept to a value less than 5% by employing dry N_2 purging to eliminate the water vapor in the air atmosphere.

We performed the SHG experiments at the TELBE THz facility at ambient condition. The schematic of the measurement setup is plotted in the Supplementary Fig. 1. Multicycle THz pulses, which are both carrier-envelope phase-stable and linearly polarized with tunable frequency, were generated in an undulator from relativistic electron bunches. In our measurements, the central frequency of the THz source was separately tuned to 0.5 THz, 0.68 THz and 1.0 THz with a repetition rate of 100 kHz. Two bandpass filters centered at the fundamental frequency with 20% bandwidth were used to suppress the background THz SHG signal. A wire-grid polarizer was positioned before the sample to calibrate the polarization state of the fundamental frequency, and the linear polarization purity of the incident terahertz wave is estimated to be around 1000. The THz radiation was focused on the metamaterial sample at normal incidence. After the sample, another wire-grid polarizer was used to block the co-polarized component of the emitted THz signal, followed by a bandpass filter centered at the generated harmonics. In the co-polarization spectrum measurement, the wire-grid polarizer after the sample was set to block the emitted cross-polarized THz signal, and another bandpass filter centered around the SHG was added. With this setup, the signal-to-noise ratio of the SHG detection was improved with the transmitted fundamental THz pump significantly suppressed, leading to the weak fundamental signal. The EOS technique was employed to measure the THz field, using a 2.0 mm thick ZnTe crystal. A commercial Ti:sapphire femtosecond laser with a central wavelength of 805 nm was synchronized with the TELBE source for EOS. The 2.0 mm thick ZnTe crystal only allows the detection of the THz field below 2.4 THz, which motivates our choice of the highest fundamental frequency at 1.0 THz, corresponding to a SHG of 2.0 THz.

In the low temperature measurement, the sample holder was placed in a liquid helium cryostat capable of cooling the sample down to 4 K. In our experiment, the temperature was tuned from 20 K to 300 K.

To study the dependence of the SHG conversion efficiency on the pumping field, a set of three wire-grid polarizers was placed before the sample. By fixing the first and last polarizers and

rotating the one in between, the amplitude of the pumping THz field could be tuned from 11% to 100% of the maximum field value.

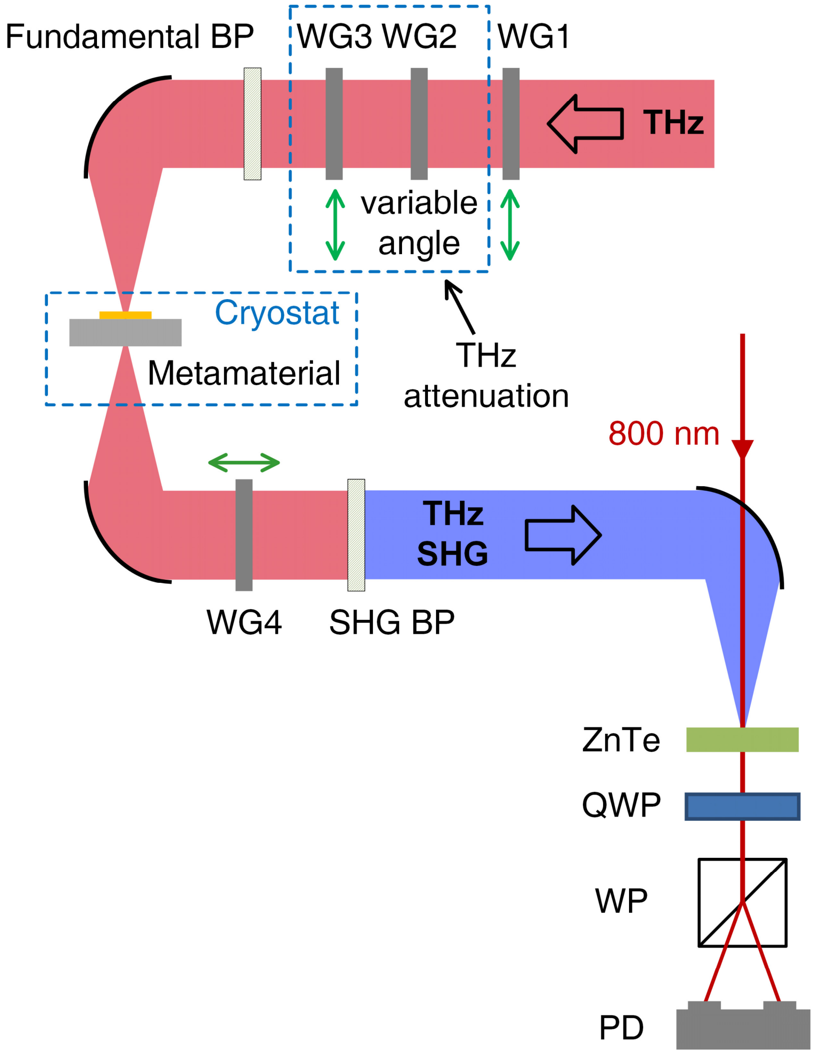

Supplementary Figure 1: Experimental set-up for the THz SHG measurement. WG: wire grid polarizer; BP: bandpass filter; QWP: quarter wavelength plate; WP: Wollaston Prism; PD: photodiode detector. The green arrows mark the state of the polarizers.

Supplementary Note 1: Theoretical model

Impact ionization provides a large number of free electrons with high pondermotive energy. The initial motion of the free electrons in the substrate can be described by a simplified Drude model as

$$m^{*}\ddot{r} = qE_0\cos(\omega t), \qquad (1)$$

where m^* is the effective electron mass, r is the displacement from the equilibrium position, q is the elementary charge, E_0 is the amplitude of the local electric field, ω is the angular frequency, and t is time. The electron collision term in the typical Drude model is omitted because the process of impact ionization occurs within a half cycle of the electromagnetic period, where the scattering is usually negligible[1,2]. By solving Supplementary Equation (1), the free electron transient velocity v_0 becomes

$$v_0 = \frac{qE_0}{m^{*}\omega}\sin(\omega t), \qquad (2)$$

and the effective value of the pondermotive energy $<U_{kin}>$ can be obtained as

$$\langle U_{kin}\rangle = \frac{1}{2}m^{*}\langle v_0\rangle^2 = \frac{q^2E_0^2}{4m^{*}\omega_0^2}, \qquad (3)$$

where $<v_0>$ is the effective velocity value in one electromagnetic period. Considering a peak electric field of 98.9 kV cm^{-1} for the pump at 0.68 THz, the pondermotive energy of the high purity silicon substrate can be calculated as 0.90 eV, which is smaller than the silicon bandgap (1.12 eV) and its threshold for impact ionization (1.1 eV)[3]. When the metamaterial is incorporated on the silicon substrate, a moderate 15 times enhancement on the local electric field leads to a pondermotive energy of 202.6 eV, which is sufficiently high to stimulate impact ionization and generate new carriers. The electric field is estimated based on the simulated results as presented in the following Supplementary Fig. 3, which is roughly half strength of the maximum.

With the charged carriers located in a strong magnetic field, the nonlinear Thomson scattering can thus be excited. The interaction between the drifting electrons and the magnetic field lasts during the entire electromagnetic wave pulse, and the electron-electron collisions in solids need to be considered, which are usually neglected in vacuum electrons. When considering the magnetic force, the free electron motion in the substrate is thus described with a modified Drude model as

$$m^{*}\ddot{r} + m^{*}\gamma\dot{r} = qE_0e^{-i\omega t} + qv\times B_0e^{-i\omega t} + c.c., \qquad (4)$$

where γ is the electron collision rate, v is the velocity of the free electrons when scattering is taken into account, B_0 is the amplitude of the local magnetic field, and *c.c.* is the complex conjugate term. Considering the relation $v = i\tilde{\mu}_e E_0 e^{-i\omega t}$, where $\tilde{\mu}_e$ is the free electron complex mobility, and the imaginary unit indicates the π/2 phase difference between the electric field and the drift velocity, Supplementary Equation (4) evolves as

$$m^*\ddot{r} + m^*\gamma\dot{r} = qE_0 e^{-i\omega t} + iq\tilde{\mu}_e E_0 \times B_0 e^{-i2\omega t}. \qquad (5)$$

For the sake of clarity, the complex conjugate term is eliminated in Supplementary Equation (5). The second-order term arising from the magnetic force represents the nonlinear Thomson scattering process, which is at the origin of the SHG. By assuming a displacement with the general solution $r = r_\omega e^{-i\omega t} + r_{2\omega} e^{-i2\omega t}$, we solved the differential equation, and the solution is

$$r_\omega = -\frac{qE_0}{m^*(\omega^2 + i\omega\gamma)}, \qquad (6a)$$

$$r_{2\omega} = -\frac{q\tilde{\mu}_e E_0 B_0}{m^*(2\omega\gamma - i4\omega^2)}. \qquad (6b)$$

The orientation of the first-order displacement is directed along the local electric field, while that of the second-order one is dictated by the resulting local magnetic and electric fields. Considering the local fields are not uniformly distributed and the polarization density is the density of the dipole moments, the second-order polarization ($P_{2\omega}$) can be obtained as

$$P_{2\omega} = \frac{q}{V_{sub}} \int_{V_{sub}} n_e r_{2\omega} dV, \qquad (7)$$

where V_{sub} is the volume of substrate where the nonlinear Thomson scattering occurs, n_e is the local density of the free electrons in the substrate, including the newly-generated ones by impact ionization, and dV is the symbol of the volume differential. We assume an average M and N times enhancement of the local electric and magnetic fields (induced by the metamaterial resonance) with respect to the incident fields. As such, Supplementary Equation (7) evolves into

$$P_{2\omega} = -\frac{q^2 MN \langle n_e \rangle \langle \tilde{\mu}_e \rangle E_{inc}^2}{c_0 m^*(2\omega\gamma - i4\omega^2)}, \qquad (8)$$

where $<n_e>$ and $<\tilde{\mu}_e>$ are average density and complex mobility of the free electrons respectively, E_{inc} is the amplitude of the pump THz field, c_0 is the speed of light in vacuum. The effective

second-order susceptibility, $\chi_{eff}^{(2)}$, can be then expressed as

$$\chi_{eff}^{(2)} = -\frac{q^2 MN \langle n_e \rangle \langle \tilde{\mu}_e \rangle Z_0}{m^* (2\omega\gamma - i4\omega^2)}, \qquad (9)$$

where Z_0 is the free space impedance.

Supplementary Equations (8) and (9) explicitly describe the second-order nonlinear processes, which is largely dominated by the metamaterial resonance. The material-related parameters in the equations, including mobility, effective mass and collision frequency, are fundamental and widely existed in solids. Consequently, the nonlinear responses are independent of any specific property of the compositions, such as the inversion asymmetry mandatory in conventional second-order nonlinear materials. In other words, besides the use of THz frequencies and a silicon substrate demonstrated in our exemplary experiments, the proposed mechanism of second-order nonlinearity can be extended, in principle, to almost all solids.

We note that the proposed metamaterials based on the split-ring resonators present very strong anisotropy, leading to the fact that there is only one scenario valid for the SHG: the x polarized component of the fundamental pump generates the y polarized second-harmonic wave. In that case, most components in the nonlinear susceptibility tensor are zero except the dominant one $\chi_{yxx}^{(2)}$. As the metamaterials offer very high design freedom, and their nonlinear responses can be artificially tailored, one could surely achieve different combinations of the pump and SHG polarization states, resulting in more complex nonlinear susceptibility tensor and much richer application potentials.

Supplementary Note 2: Strong-field THz transmission spectra

The time-domain terahertz waveform directly generated from the optical rectification of the OH1 crystal is mapped out as shown in Supplementary Fig. 2a, with the peak amplitude of 1.59 MV cm^{-1}. The frequency-domain spectrum (Supplementary Fig. 2b), obtained by Fourier transform of the time-domain pulse, presents a broad frequency band. Given only a small portion of the generated THz field resonates with the metamaterial, the original pulse was then spectrally filtered by a bandpass filter centered at 0.8 THz, where is the amplitude peak closest to the resonance of the metamaterial. The resulting multi-cycle THz pulse is shown in Supplementary Fig. 2c, corresponding to the frequency-domain spectrum shown in Supplementary Fig. 2d. The peak amplitude of the incident THz electric field is 22.1 kV cm^{-1}. Although weaker than that of

the TELBE source, it is sufficiently strong to excite the impact ionization with the resonance of the SRR. The newly-populated free electrons increase the conductivity of the silicon, causing the redshift of the resonance frequency, and the increase and broadening of the transmission, as shown in the measured spectra (Fig.2a in the main text). At the lowest pump amplitude of 5.5 kV cm^{-1}, the same enhancement factor of 15 leads to the ponderomotive energy of 0.63 eV, smaller than the silicon bandgap. We thus believe the impact ionization is not significant.

In the meantime, we spectrally filtered the THz pulse with a bandpass filter centered at 1.6 THz, where the peak amplitude is higher than that of 0.8 THz. An amplitude peak of 71.9 kV cm^{-1} is obtained in the resulting multi-cycle THz pulse (Supplementary Fig. 2e), which is comparable to that of the TELBE source. The reported peak amplitudes of the table-top THz sources reach 83 MV cm^{-1}, over one order of magnitude stronger than the one in our setup[4,5]. They are expected to provide stronger multi-cycle narrow-band THz pump than the TELBE source with the proper bandpass filters.

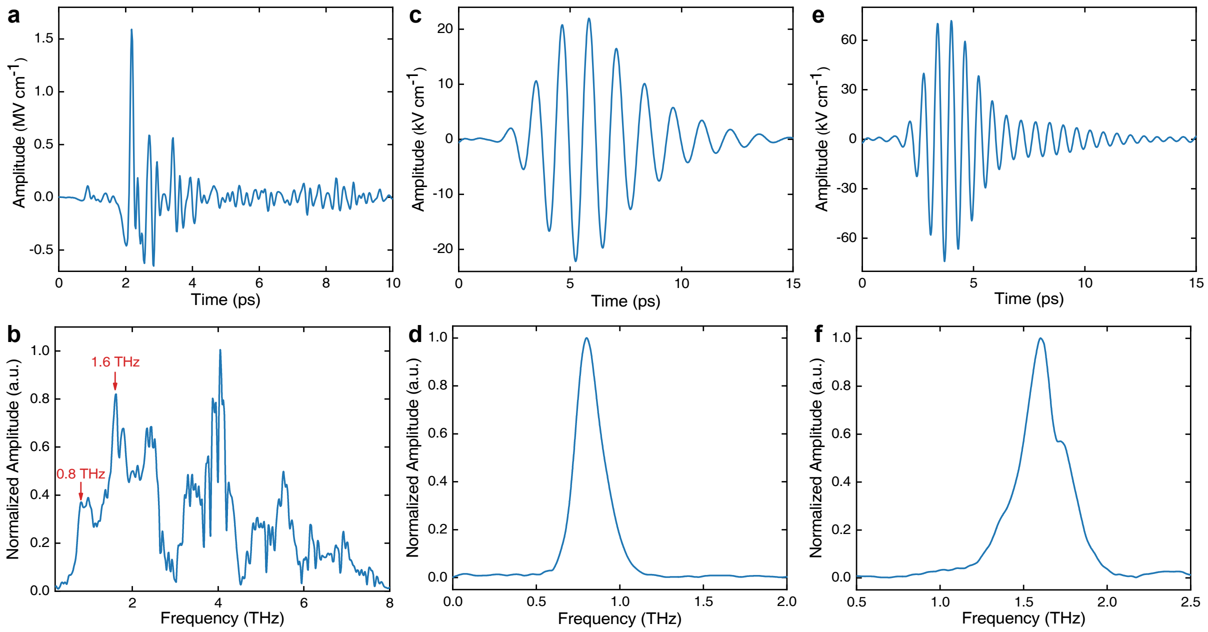

Supplementary Figure 2: Measured THz time-domain spectra and their corresponding frequency spectra from the table-top strong-field THz time-domain spectroscopy. a, The time-domain spectrum of the original THz pulse, and **b**, its amplitude frequency-domain spectrum. Red arrows mark the peaks at 0.8 THz and 1.6 THz. **c**, The time-domain spectrum of spectrally filtered multi-cycle THz pulse with a bandpass filter centered at 0.8 THz, and **d**, its amplitude frequency-domain spectrum. **e**, The time-domain spectrum of spectrally filtered multi-cycle THz pulse with a bandpass filter centered at 1.6 THz, and **f**, its amplitude frequency-domain spectrum. All three frequency-domain

spectra are normalized to their maximum.

Supplementary Note 3: Field distributions and estimation of the interaction thickness

The simulated magnetic and electric field distributions of the metamaterial at 0.68 THz are revealed in Supplementary Fig. 3. The enhanced magnetic field perpendicular to the surface of the metamaterial reaches a maximum value 57.4 times as intense as the incident field. The local electric field in the x direction presents a maximum value 32.1 times stronger than the pump. With the resulting enhanced magnetic and electric fields, an intense Lorentz force arises, as shown in Fig. 2b of the main text. It should be noted that the distributions are simulated under a weak field condition, and the resonance shift caused by impact ionization is not considered.

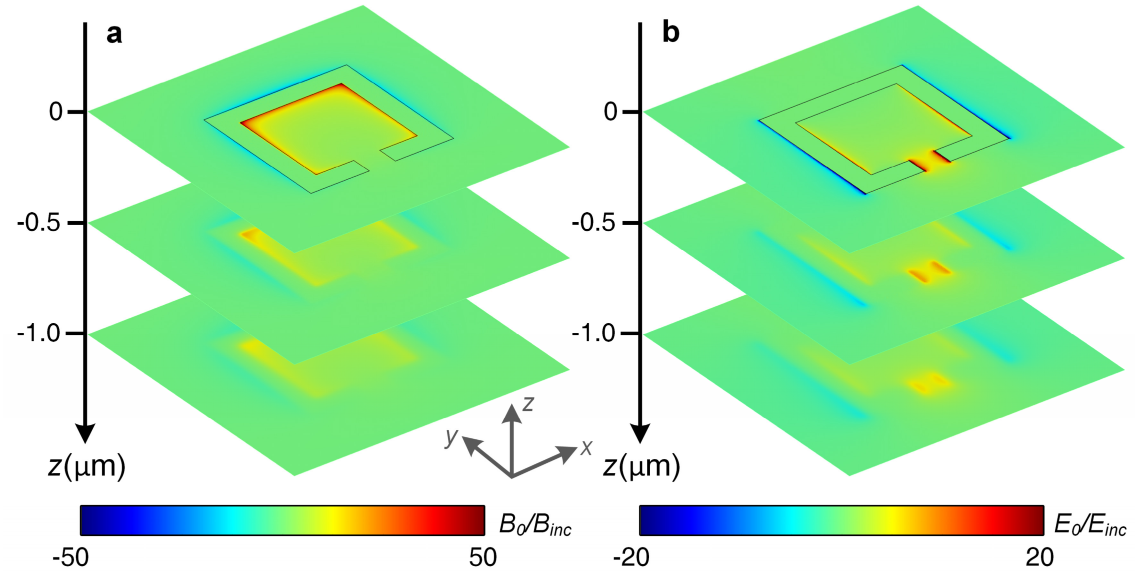

Supplementary Figure 3: Simulated field distribution of the metamaterial at 0.68 THz. a, The z component of the magnetic field, B_0; **b,** the x component of the electric field, E_0, normalized to the corresponding amplitudes of the incident fields (B_{inc} and E_{inc}).

The thickness of the interaction layer is important in evaluating the effective nonlinear susceptibility. As it is technologically difficult for the direct measurements, we carefully estimate the thickness based on the simulated electric and magnetic field distributions. Starting with the Supplementary Equation (9), the effective second-order susceptibility of a layer at the distance z_1 beneath the substrate-SRR interface can be approximately expressed as

$$\chi_{eff}^{(2)}\left(z_1\right) \propto \left\langle n_e \right\rangle MN . \qquad (10)$$

For simplicity, the influences of other parameters, such as the mobility and the collision rate of free electrons, are omitted because their variations at different z coordinates are relatively weak, less than one order of magnitude. Impact ionization dominates the free electron density, which follows the relation[6,7].

$$\langle n_e \rangle \propto e^{-\frac{E_{th}}{E_0}} = e^{-\frac{E_{th}}{ME_{inc}}}, \qquad (11)$$

where E_{th} is the characteristic field of impact ionization with the value of 1.7 MV cm^{-1}. It shows that the effective susceptibility is dominated by the strength of the local electric and magnetic fields, which dramatically decay inside the substrate as shown in the Supplementary Fig. 3.

To quantitatively compare the nonlinear contributions of the layers at different z_1 coordinates, we define a ratio K_d as

$$K_d(\mathrm{z}_1) = \chi_{eff}^{(2)}(\mathrm{z}_1) / \chi_{eff}^{(2)}(z_0{=}0\mu m). \qquad (12)$$

The simulated field distributions show that at z_0=0 μm (the substrate-SRR interface), the maximum values of $(M\times N)_{max}$=265, M_{max}=32.1, and N_{max}=57.4 can be achieved and those at z_1=0.5 μm are $(M\times N)_{max}$=32.6, M_{max}=7.2, and N_{max}=21.5. Due to the non-uniformly distribution of the local fields, the $(M\times N)_{max} \neq M_{max}\times N_{max}$. At the maximum pump of 98.9 kV cm^{-1}, $K_d(0.5\ \mu m)$ can be calculated as 0.019, meaning that the contribution of the layer at 0.5 μm to the overall nonlinear responses is much weaker than that at 0 μm and can be neligible. For the layer at deeper position, for example, at z_2=0.6 μm, it leads to an even weaker $K_d(0.6\ \mu m)$= 9.7×10^{-3} with the values of $(M\times N)_{max}$=25.1, M_{max}=6.1, and N_{max}=19.0, showing the deeper region plays very minor roles in the nonlinear response. We thus believe the 0.5 μm thick interaction vicinity is a sound and very tolerant estimation. With the decreasing pumping field, the effective thickness would be even thinner.

Supplementary Note 4: Characterization of the measured SHG at THz frequencies

The measured spectrum of the fundamental THz pump from TELBE was plotted in Supplementary Fig. 4a. The emitted SHG spectrum of the silicon-based sample was extracted by means of Fourier-filtering, which was cross-polarized with respect to the fundamental field (see Supplementary Fig. 4b). The pulse width of the SHG is much wider than the one at the fundamental frequency, due to multiple reflections in the substrate. The measured interval time of 11.0 ps between the reflected pulses is in good agreement with the theoretical value of 11.4 ps, calculated assuming a silicon permittivity of 11.7 and a thickness of 500 μm for the silicon substrate. We

integrate the intensities of the fundamental wave and second harmonics over the full pulse duration, and the intensity conversion efficiency of the SHG η_I can be accurately extracted as

$$\eta_I = \frac{\int_{t_{2\omega}} E_{2\omega}^2(t)\,dt}{\int_{t_\omega} E_{inc}^2(t)\,dt}, \qquad (13)$$

where $t_{2\omega}$ and t_ω are the pulse durations of the SHG and the fundamental wave respectively, while $E_{2\omega}(t)$ and $E_{inc}(t)$ are the amplitudes of the SHG and the pump at time t, respectively. The intensity conversion efficiency of the silicon-based metamaterial sample was calculated as 1.2×10^{-7}, corresponding to the amplitude conversion efficiency of 3.5×10^{-4}.

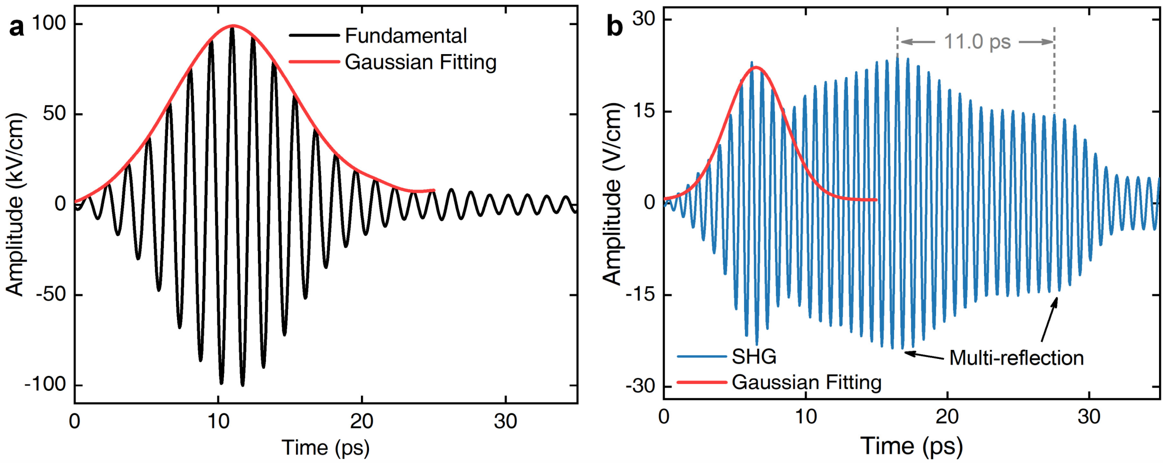

Supplementary Figure 4: Measured time-domain spectra. a, The fundamental THz pump; b, the generated second-harmonic radiation. The pulse envelopes are extracted with a Gaussian Fit. The SHG is cross polarized with respect to the fundamental wave.

The full-widths at half maximum (FWHM) of the fundamental and SHG fields can be obtained with a Gaussian Fit, which are 7.2 ps and 3.2 ps, respectively. Conventionally, the SHG pulse is expected to be $\sqrt{2}$ times shorter than that of the pump wave. In our measurements, however, the shortening effect on the SHG pulse is more evident, being $2.25\approx\sqrt{5}$ times shorter. This is because the proposed second-order nonlinear processes can only occur at an incident electric field exceeding the threshold of impact ionization, leading to an effective pump pulse width narrower than the actual value and to a much shorter SHG pulse. The measured results fully support our theory and the related fundamental role of impact ionization.

To characterize the SHG polarization state, we experimentally examined the emitted spectrum

of the sample for a polarization parallel to that of the incident field. When measuring the co-polarized spectrum, an extra bandpass filter centered around the SHG were added to better suppress the transmitted fundamental pump. The cross-polarized spectrum was also measured with the same setup for comparison. As shown in Supplementary Fig. 5, the cross-polarized SHG is over two orders of magnitude stronger than that in the co-polarized one. The extra bandpass filter leads to a very weak transmitted fundamental signals, and that in the cross-polarized spectrum is barely observable. The degree of linear polarization (DOLP) parameter, $D_{2\omega}$, was introduced to evaluate the polarization state as follows,

$$D_{2\omega} = \frac{I_{cross} - I_{co}}{I_{co} + I_{cross}}, \qquad (14)$$

where I_{cross} and I_{co} represent the intensities of the SHG for a cross- and co-polarized state with respect to the incident field, respectively. The extracted 99.0% value for $D_{2\omega}$ at the doubled frequency of 1.36 THz fully demonstrates the cross-polarized nature of the SHG induced by the nonlinear Thomson scattering, perfectly agreeing with our theoretical model. We believe the secondary peak close to 1.36 THz comes from a leakage of the THz pump. Even taking it into consideration, the $D_{2\omega}$ achieves a value of 92.3%, confirming the dominate role of the cross-polarized component in the SHG.

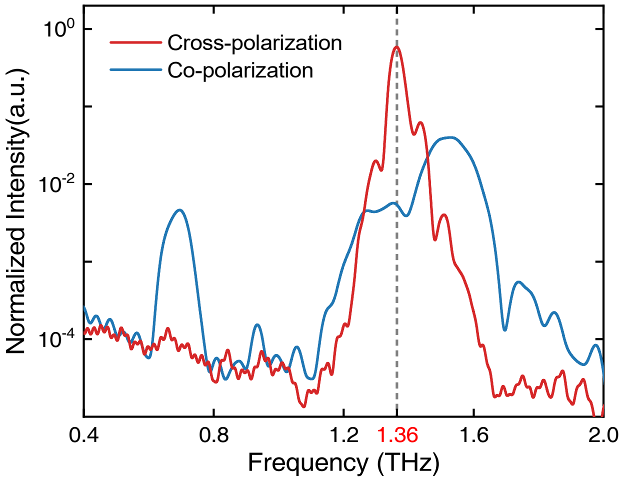

Supplementary Figure 5: Measured frequency-domain spectra emitted by the metamaterial sample in cross- and co-polarized states with respect to the fundamental polarization. The grey dashed line marks the doubled frequency at 1.36 THz. The intensity is normalized to the peak intensity of the SHG shown in Fig.2c in the main text, which was measured with one filter after the sample.

Supplementary Note 5: Influence of the temperature on the SHG intensity

Many of the factors in Supplementary Equation (9) are sensitive to temperature. For example, the total density n_e of free electrons is given by the combination of the intrinsic free electron density and the nonequilibrium electron density induced by impact ionization, and its temperature dependence can be described as[8]

$$n_e(T) = n_i(T)(1+\alpha_{ii}(T)) \approx n_i(T)\alpha_{ii}(T), \qquad (15)$$

where $n_i(T)$ is the intrinsic free electron density at a temperature T in Kelvin. $\alpha_{ii}(T)$ is impact ionization rate, defined as the number of new electrons generated by a solitary initial carrier, which is much larger than one in our case, thereby rendering the approximation valid. Impact ionization is somewhat affected by cooling, with previous literature suggesting that $\alpha_{ii}(T)$ increases by slightly less than one order of magnitude with the temperature dropping from 500 K to 50 K[9]. The intrinsic density of free electrons as a function of temperature is expressed as[10]

$$n_i(T) \propto T^{\frac{3}{2}} e^{-\frac{E_g}{2k_B T}}, \qquad (16)$$

where E_g is the silicon bandgap and k_B is the Boltzmann constant. From Supplementary Equations (15) and (16), we see that cooling down the silicon-based metamaterial results in a reduction in the intrinsic equilibrium free electron density dominated by exponential activation, which is not substantially compensated by the increasing impact ionization rate. Further, the decrease of the total free electrons together with the low temperature would improve the mobility $<\tilde{\mu}_e>$ of silicon and the quality factor of the metamaterial resonance. In particular, for silicon with low doping concentration, which is our case, the temperature dependence of the dc mobility μ_{e0} roughly follows the relation[10]

$$\mu_{e0} \propto T^{-\frac{3}{2}}. \qquad (17)$$

Finally, the electrons produced by impact ionization lead to an increase of conductivity and so weaken the resonant strength of the metamaterial. This negative correlation between free electron density and resonance quality means that the fewer electrons generated at lower temperature improve the resonant strength and local fields of metamaterial.

Despite putting the metamaterial sample into a cryogenic environment favors most factors in the physical model that would improve the effective second-order susceptibility, they do not entirely overcome the exponential decay of the intrinsic free electron density in high purity silicon.

The upshot is that the experimental SHG shown in Fig. 2d of the main text decreases monotonically with decreasing temperature. We can fit the data to the phenomenological form

$$\chi_{eff}^{(2)}(T) \propto n_i^{C_T}(T) \propto e^{-\frac{C_T E_g}{2k_B T}}, \qquad (18)$$

where C_T is a coefficient accounting for all factors which counteract the decrease in the intrinsic carrier density in Supplementary Equation (9). With the measured data, the coefficient C_T for the designed metamaterial sample is fitted as 0.06. The small value of C_T is a proof of the positive influence originating from the physical properties benefiting from the low temperature. Excluding the performance improvements at low temperature due to the mobility, impact ionization rate, and resonant strength of metamaterial, C_T would be close to 1.0.

It should be noted that in our case, the impact ionization-induced electron-hole pairs are in an *unbound* state, which could interact with the local electric and magnetic fields. At very low temperature, below 30 K for silicon[11], the populated electron-hole pairs may further form *bound* neutral excitons, when the THz electric field that would otherwise ionize the excitons is absent. After the THz pulse has passed, recombination would eventually lead to luminescence which increases with decreasing temperature. Such luminescence experiments, previously used to examine impact ionization in semiconductors[1], are therefore complementary to ours, which are sensitive to *unbound* electrons, the number of which grows with temperature.

Supplementary Note 6: Amplitude dependence of the SHG on the pump field

The SHG amplitude dependence on the pump field is not quadratic due to the joint mechanism of impact ionization and nonlinear Thomson scattering. The dependence of the total electron density on the pump field is[8,12]

$$n_e(E_{inc}) = n_i + \Delta n_e(E_{inc}), \qquad (19)$$

where

$$\Delta n_e(E_{inc}) \propto n_i e^{-\frac{E_{th}}{ME_{inc}}}, \qquad (20)$$

$\Delta n_e(E_{inc})$ is the nonequilibrium electron density induced by the impact ionization at the pump field E_{inc}, and n_i is the intrinsic free electron density of the substrate. With an increasing pump field, more and more electrons are ionized, leading to a shift of the metamaterial resonance. Meanwhile, more electrons increase the scattering rate and degrade the mobility. For such reasons, the behavior

of the SHG amplitude differs at different pump fields, and can be divided into three regimes.

In the first regime, the pump field is slightly over the threshold of impact ionization, and the new electrons starts to be ionized, but the density is not high enough to clearly affect the metamaterial resonance and scattering. The THz SHG amplitude and the nonlinear susceptibility go as $E_{2\omega} \propto P_{2\omega} \propto E_{inc}^2 n_e(E_{inc})$ and $\chi_{eff}^{(2)} \propto n_e(E_{inc})$, respectively, meaning that the effective nonlinear susceptibility grows with a stronger illumination.

When the pump field keeps increasing, the free electron density is high enough to degrade the mobility and cause significant variations in the conductivity of the substrate. In particular, the latter further shifts the SRR resonance and weakens the local field enhancement. The growth rate of free electrons decreases as well, despite the total density increases. In this case, an increasing intensity of the fundamental pump results in the decay of the effective susceptibility.

In the final region where the pump field is very strong, the substantial numbers of free electrons generated at the earlier stage of the carrier multiplication may undergo a significant amount of the electrons scattering. The scattering decelerates the electrons and reduces their ability to gain kinetic energy, which eventually suppresses the impact ionization events[1,7]. The total free electron density thus remains constant due to the saturation of the impact ionization, leading to a steady state for both mobility and SRR resonance. Therefore, the SHG amplitude shows a quadratic dependence on the pumping field as $E_{2\omega} \propto P_{2\omega} \propto E_{inc}^2$, and the effective nonlinear susceptibility becomes constant, as in natural materials.

In the measurements, the effective susceptibility is extracted from the conversion efficiency. A classical expression for the SHG intensity in a nonlinear medium is[13,14]

$$I_{2\omega} = \frac{(2\omega)^2}{8c_0^3\varepsilon_0\varepsilon_\omega\sqrt{\varepsilon_{2\omega}}}\left|\chi_{eff}^{(2)}\right|^2 d^2 I_{inc}^2 \cdot \mathrm{sinc}^2\left(\frac{\Delta kd}{2}\right), \qquad (21)$$

where $I_{2\omega}$ is the SHG intensity, I_{inc} is the incident wave intensity, ε_0 is the vacuum permittivity, k_ω and $k_{2\omega}$ are the wavenumbers of the fundamental and second-order waves, $\Delta k=k_{2\omega}-2k_\omega$, ε_ω and $\varepsilon_{2\omega}$ are the relative permittivities of the nonlinear medium at frequency ω and 2ω, respectively, and d is the material thickness. In our silicon-based samples, the value of the permittivity at the fundamental and doubled THz frequencies is the same. With Δk=0 and sinc($\Delta kd/2$)=1, the metamaterial samples can be thought as equivalent to a thin slab featuring a phase-matched second-order nonlinearity. This equivalence can also be understood in the following way. The SHG

intensity in the metamaterial reaches a maximum under a linearly polarized, normally incident field. The commonly-used methods to achieve a phase matching condition in nonlinear optics, such as an oblique incident field and/or configuring the impinging polarization state, cannot improve the metamaterial nonlinear response, since this may have a negative impact on the resonance. Given the fact that bulk nonlinear materials achieve the maximum SHG intensity under phase-matching, it is appropriate to assume an equivalent condition for the metamaterial.

With some basic mathematical operations, we derived the effective nonlinear susceptibility as

$$\left|\chi_{eff}^{(2)}\right| = \frac{2c_0\sqrt{\eta_{I,eff}\varepsilon_{2\omega}}}{\omega d E_{inc}}, \qquad (22)$$

where $\eta_{I,eff}$ is the effective intensity conversion efficiency for the SHG. Since the nonlinear process originates from the metamaterial resonance, the effective volume of the material should be confined in the area of the SRR times a 0.5 μm thick layer in our case. The effective intensity conversion efficiency can thus be derived as $\eta_{I,eff}=\eta_I/A_{eff}$, where A_{eff} is the area duty cycle of the SRR in the unit cell. By substituting the corresponding parameters, the effective nonlinear susceptibility can be readily worked out. At the maximum pump, the parameters are $\eta_I=1.22\times10^{-7}$, $A_{eff}=0.269$, $\varepsilon_{2\omega}=11.7$, $\omega=2\pi\times0.68\times10^{12}$ rad/s, $E_{inc}=98.9$ kV cm^{-1}, and $d=0.5$ μm, and we thus obtain $\chi_{eff}^{(2)} = 6.5\times10^4$ pm V^{-1}.

Supplementary Note 7: Numerical Modeling of the THz SHG in metamaterial

In the numerical simulations of the THz SHG, a slab-shaped volume with different free electron densities was modelled beneath the SRR in the substrate, so as to mimic the carrier generation caused by impact ionization (see Supplementary Fig. 6a). The geometry of the slab is dictated by the field distributions in the weak illumination regime as shown in Supplementary Fig. 3, which is slightly larger than the SRR. The thickness of the slab, t_{eff}, decreases with increasing carrier density due to the skin effect, following the relation $t_{eff} \propto \sigma_0^{-1/2}(n_e)$. We plot the simulated metamaterial transmission spectra for a free electron density varying from 4×10^{15} cm^{-3} to 10^{18} cm^{-3} (see Supplementary Fig. 6b). It can be found that as the free electron density grows, the resonance decays with a clear redshift, leading to a different response of the metamaterial for the 0.68 THz driving field.

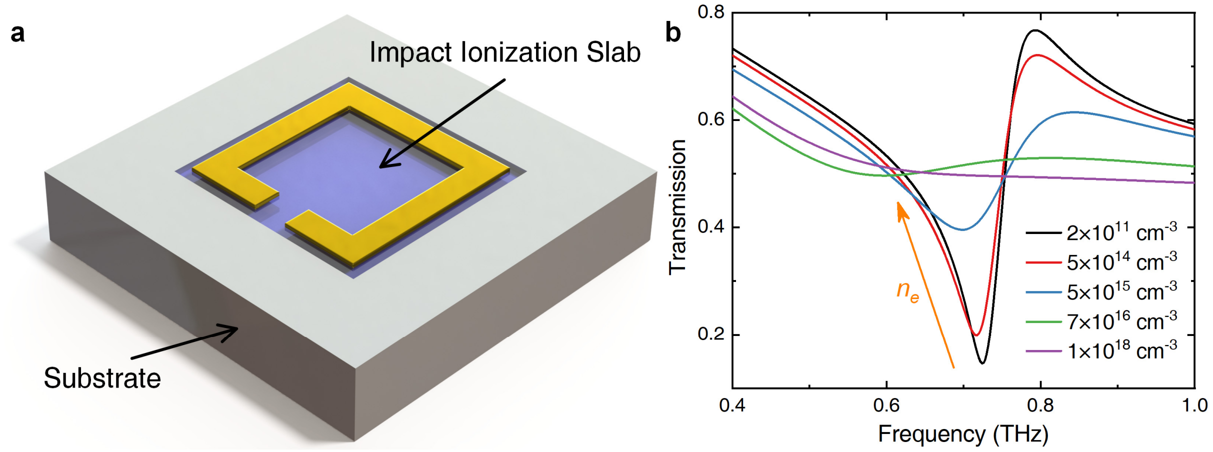

Supplementary Figure 6: Influence of impact ionization on the metamaterial resonance. a, A conductive slab under the SRR represents the carrier generation in the substrate induced by impact ionization; **b**, the metamaterial transmission spectra for a series of free electron densities in the slab.

By taking into consideration the magneto-electric coupling, the time-domain response of the metamaterial was then simulated. A Gaussian pulsed plane wave at the fundamental frequency of 0.68 THz impinged from the top with a polarization orthogonal to the SRR gap (along the x axis). As revealed in Supplementary Fig. 7a, we evaluated a cross-polarized transmission spectrum of the metamaterial in the time domain with $n_e = 10^{18}$ cm^{-3}, and a 1.36 THz wave could be extracted by the Fourier-filtering, indicating THz SHG. The frequency spectrum in Supplementary Fig. 7b, Fourier-transformed from the time-domain, more explicitly shows the presence of SHG. As expected, only the fundamental THz frequency is observed from the silicon substrate with the same conductive slab, and the metamaterial sample under a pump polarization parallel to the gap (along the y axis). We also observed the co-polarized transmission spectrum, as shown in Supplementary Fig. 7c, and found only negligible SHG, corresponding to a DOLP close to unity, perfectly agreeing with both theory and measurements. Our simulations properly capture the key experimental features, including polarization sensitivity and the SHG polarization state.

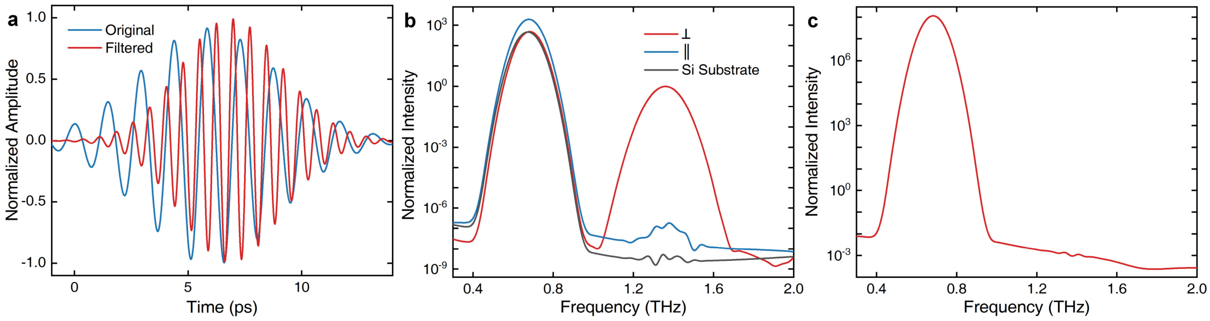

Supplementary Figure 7: Simulated metamaterial nonlinear spectra. a, Original and filtered time-domain metamaterial spectra (cross-polarized with respect to the fundamental wave), normalized to the corresponding maximum; **b**, cross-polarized metamaterial frequency-domain spectra for the polarization of the fundamental wave orthogonal (along the x axis) and parallel (along the y axis) to the SRR gap; the case of a silicon substrate incorporating an equivalent conductive slab is also shown; **c**, co-polarized metamaterial frequency-domain spectrum for the polarization of the fundamental wave orthogonal to the SRR gap (along the x axis). In **b** and **c**, the spectra are normalized to the SHG peak intensity.

Supplementary Note 8: Comparisons with nonlinear Thomson scattering in relativistic electrons

The occurrence of the nonlinear Thomson scattering requires that the contributions of the magnetic and electric fields of the light on the electron motions should become comparable. Considering a free electron in vacuum interacting with the electromagnetic field at the angular frequency ω, its peak velocity is $v_e=qE_{inc}/(m_0\omega)$, where m_0 is the rest electron mass. The ratio of its magnetic and electric forces, a_0, can thus be obtained as

$$a_0 = \frac{qv_eB_{inc}}{qE_{inc}} = \frac{v_e}{c_0} = \frac{qE_{inc}}{m_0\omega c_0}, \tag{23}$$

where the relation $B_{inc}=E_{inc}/c_0$ is applied. It indicates that the nonlinear Thomson scattering occurs in the electrons with velocities approaching the speed of light, which was only satisfied in electrons in vacuum. The dimensionless parameter a_0, also known as normalized vector potential, is widely used to evaluate incident electromagnetic field strength for the nonlinear Thomson scattering[15-18].

In our solid-state metamaterial, the large contribution of the magnetic force is achieved by the locally enhanced magnetic field instead of pushing the drift motions of electrons to the relativistic limit, which makes the occurrence of the nonlinear Thomson scattering in the condensed matters possible. Similarly, we compare the local magnetic force with the electric force from the incident field, and the ratio is

$$a_{meta} = \frac{qvB_0}{qE_{inc}} = \frac{qE_{inc}MN}{m^{*}c_0(\omega+i\gamma)}, \tag{24}$$

where M and N are average enhancement times of the local electric and magnetic fields, m^* is the effective electron mass, and γ is the electron collision rate. It shows that the local fields enhancements together with the unique band structure of solids significantly relax the intensity of the pump. Taking the parameters in our experiments at the incident electric field of E_{inc}=32.5 kV cm^{-1}, including ω=2π×0.68×10^{12} rad/s, $m^*=0.26m_0$, γ=2π×1.18×10^{12} rad/s and $(M\times N)_{max}$=265, we obtain the amplitude of a_{meta} as 0.23 which is 509 times larger than that of a_0 as 4.5×10^{-4}. It suggests that an over 5 orders of magnitude stronger terahertz source is needed to reach the similar strength of the nonlinear Thomson scattering for the rest electron in vacuum, which is currently unrealistic. In practice, intense lasers at near infrared and visible frequencies are available to reach large normalized vector potential.

Given the commonly-used femtosecond lasers at the wavelength of 800 nm[15], the same normalized vector potential of 0.23 asks for the amplitude of peak electric field as high as 9.2 GV cm^{-1}, corresponding to the intensity of 1.1×10^{17} W cm^{-2}. This level of intensity is over 10 orders of magnitude stronger than that of the terahertz source we used and will severely damage most solids. In addition, the charged carrier density in solids is usually orders of magnitude higher than that the free electrons in vacuum, further boosting the efficiency of the nonlinear responses. The slow motions of the electrons in condensed matters avoid tons of the complex side effects in the relativistic regime, such as the increase of electron mass, the Doppler shift of the scattered light frequency, and bremsstrahlung[17,18], which provide a much purer platform for further research and application of the Thomson scattering mechanism.

As suggested in our theoretical model, many solid-state material properties, including the crystalline lattice, the bandgap structure and quantum states of the carriers, together with the artificial design of the metamaterial offer myriad new freedoms to tailor and control the Thomson scattering processes. A simple process of ion implantation modulates the temporal coherence and power dependence of the nonlinear emission, and completely reverses the temperature dependence, as demonstrated in the main text. The frequency response can be conveniently tailored by scaling the metamaterial structure as shown in the Supplementary Note 12. None of these functions can be achieved in the conventional relativistic media such as plasma and gas. We thus anticipate lots of singular possibilities brought about by the nonlinear Thomson scattering in solids are largely unexplored. For example, incorporating this metamaterial-based approach with the massless Dirac matters will result in a large effective value of the normalized vector potential and very high-order

harmonic generation even under weak optical pump as indicated in Supplementary Equations (24), which may set the foundation of novel radiation sources.

Supplementary Note 9: Contribution of the surface second-order nonlinearity

Although the centrosymmetric solids, like silicon and gold, do not present bulk second-order nonlinearity, the SHG may be excited at the surface where the symmetry is broken. In the nonlinear plasmonics and metasurfaces working at near-infrared and visible regimes, the surface second-order nonlinearities of noble metal play crucial roles in the SHG[19]. However, at the terahertz frequencies, these surface contributions can be neglected. We analyze the intensity conversion efficiency of the SHG, η_I, which follows the relation[13,14],

$$\eta_I = \frac{I_{2\omega}}{I_{inc}} \propto \left|\chi^{(2)}\right|^2 d^2, \qquad (25)$$

where $I_{2\omega}$ is the SHG intensity, I_{inc} is the incident wave intensity, $\chi^{(2)}$ is the second-order susceptibility, and d is the interaction thickness. For the gold film, the surface second-order polarizations are dominated by the convective terms, which follows[20,21]

$$(\omega^2 + i\omega\gamma_m)P_{1,m} = -\varepsilon_0\omega_{p,m}^2 E_0, \qquad (26a)$$

$$(4\omega^2 + 2i\omega\gamma_m)P_{2,m} = -\frac{\omega^2}{n_m q}[(\nabla\cdot P_{1,m})P_{1,m} + (P_{1,m1}\cdot\nabla)P_{1,m}], \qquad (26b)$$

where γ_m is the electron collision rate of gold, $\omega_{p,m}$ is the plasma frequency of gold, ε_0 is the vacuum permittivity, $P_{1,m}$ and $P_{2,m}$ are the first- and second-order polarizations of gold, n_m is the free electron density of gold, E_0 is the amplitude of the local electric field. The effective second-order susceptibility of the gold surface ($\chi_m^{(2)}$) can thus be obtained with the relation

$$P_{2,m} = \varepsilon_0\chi_m^{(2)}E_{inc}^2. \qquad (27)$$

Together with the Supplementary Equation (9), we can quantitatively compare the nonlinear contributions from the surface of gold and the Lorentz force. We use literature parameters for the gold plasma frequency (1.367×10^{16} s^{-1}) and the electron collision rate (1.062×10^{14} s^{-1}) [22]. With the impact ionization, we assume the free electron concentration of silicon to be 10^{18} cm^{-3}, corresponding to silicon dc mobility of 277 cm^2 V^{-1}s^{-1} and the electron collision rate of 2.44×10^{13} s^{-1}. In that case, considering the maximum local electric and magnetic fields enhancement, we obtain the ratio $R_\chi = \left|\chi_{eff}^{(2)}\right| / \left|\chi_m^{(2)}\right|$, as 8.8, meaning the nonlinear responses from the nonlinear

Thomson scattering is stronger than those from the surface of the gold SRR. More importantly, gold as a centrosymmetric material, the surface nonlinearities only happen in one or two atomic layers at the interfaces[20,23], which is less than 1 nm thick. While the Thomson scattering is a bulk term, whose interaction thickness is 500 nm according to our estimations. With the Supplementary Equation (25), the intensity conversion efficiencies of the SHG from different origins are compared as

$$R_\eta = \frac{\eta_{eff}}{\eta_m} \propto \frac{\left|\chi_{eff}^{(2)}\right|^2 d_{eff}^{\ 2}}{\left|\chi_m^{(2)}\right|^2 d_m^{\ 2}}, \qquad (28)$$

where η_{eff} and η_m, d_{eff} = 500 nm and d_m = 1 nm are the conversion efficiency and the interaction thicknesses of the second-order nonlinearity from the nonlinear Thomson scattering and the gold surface, respectively. We thus obtain the value of R_η as 1.9×10^7, showing the contribution from the magneto-electric coupling overwhelms the one produced by the gold surface by over 7 orders of magnitude.

Supplementary Note 10: SHG with an ion-implanted silicon substrate

The linear transmission spectrum of the doped-silicon sample was first measured as depicted in Supplementary Fig. 8a, where a shallow resonance around 0.7 THz was recorded. The weak strength of the resonance is attributed to the use of the doped silicon film, which presents stronger absorptive loss in the THz regime than undoped high purity silicon.

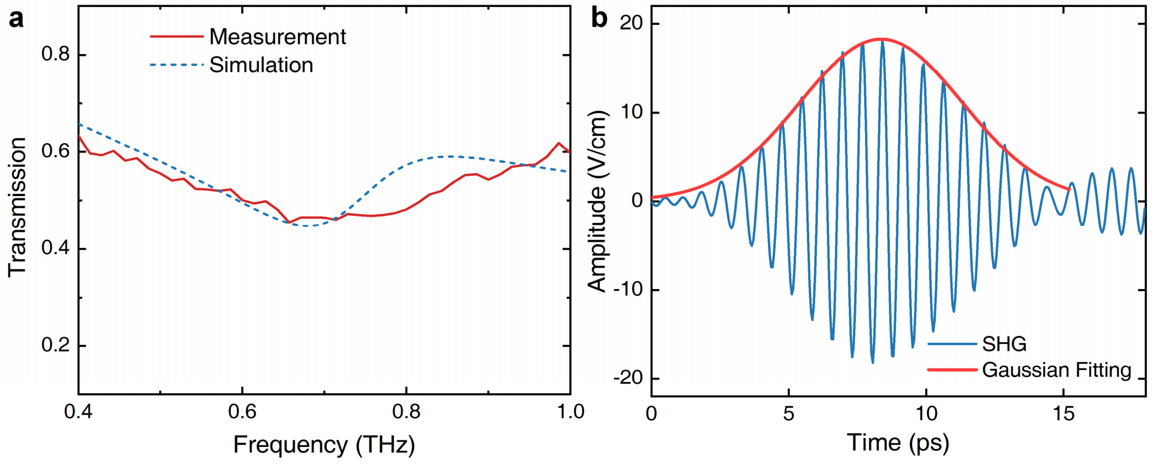

Supplementary Figure 8: Linear and nonlinear results of the doped-silicon metamaterial. a, Measured and simulated linear transmission spectra. **b**, Measured cross-polarized time-domain spectrum of THz SHG where the envelope is extracted with a Gaussian Fit.

For the nonlinear measurement with a 0.68 THz pump, the time-domain spectrum of the doped-silicon sample recorded in a cross-polarized state are plotted in Supplementary Fig. 8b. We observed THz SHG with an intensity conversion efficiency of 2.50×10^{-8}. The thickness of the doping film processed by the ion-implantation technique conditions we used can be estimated as around 0.3 μm, thinner than the 0.5 μm value for the high-purity silicon sample, and an effective nonlinear susceptibility $\chi_{eff}^{(2)}$ of the doped-silicon sample can thus be extracted as 4.9×10^{4} pm V^{-1}. Even with the same effective thickness, the doped-silicon sample still leads to a value of 3.0×10^{4} pm V^{-1}. Both values are orders of magnitude higher than those of the typical nonlinear media at optical frequencies.

From the time-domain spectrum, we extracted an FWHM of 5.1 ps for the THz SHG pulse, perfectly consistent with a $\sqrt{2}$ times shorter than the pump pulse, corresponding to the $\sqrt{2}$ times wider SHG peak in the frequency-domain shown in the inset of Fig. 4a in the main text. For the doped-silicon substrate, free electrons come from impurities and are independent from the electric field. The nonlinear response follows the rules of a conventional second-order nonlinearity, leading to the $\sqrt{2}$ times shorter SHG pulse demonstrated in our experiments.

Considering the density and complex mobility of n-doped silicon at the temperature ranging from 20 K to 300 K[10,24], we obtained the theoretical dependence of the THz SHG intensity on the sample temperature based on the Supplementary Equation (9), as shown in the Fig. 4a of the main text. At the temperature above 100 K, the majority of the doped impurities in silicon are thermally ionized, and the free electron density varies little. Mobility benefits from the low temperature due to the lower scattering rate, leading to the increase of the THz SHG intensity.

Very low thermal energy at the temperature below 100 K cannot fully ionize the impurities. In that case, the mobility first increases then decreases with the dropping temperature due to the different temperature dependence of the phonon and impurity scatterings[10,24], leading to a similar behavior of the THz SHG in both experimental and theoretical perspectives. As a room-temperature model, the effective second-order susceptibility described in the Supplementary Equation (9) does not consider the factors those only matter at cryogenic temperature, resulting in the slight variations between the theoretical prediction and measured results. For example, the fundamental and second-harmonic THz frequencies (0.68 THz and 1.36 THz) have photon energy of 2.8 meV and 5.6 meV, respectively (k_BT value at 50 K is 4.3 meV), which may help to produce

more free electrons through the impurity absorption. Ohmic loss of the SRR at resonance may locally heat the silicon and ionize more impurities as well. These extra free electrons caused by the imping THz energy result in a much lower value of the actual mobility than the theoretical one due to the higher scattering rate, thus leading to the weaker THz SHG in the measurements.

Supplementary Note 11: SHG in Sapphire-based metamaterial

We fabricated the same metamaterial structures on a c-cut sapphire substrate to demonstrate the generality of the nonlinear mechanism demonstrated here. As shown in Supplementary Fig. 9a, the measured linear transmission spectrum of the sample presents a clear resonance at 0.73 THz, which is almost the same as that of the silicon-based samples, due to a very similar permittivity. Under a 0.68 THz wave pump, the cross-polarized nonlinear spectrum is shown in Supplementary Fig. 9b. A doubled frequency signal centered at 1.36 THz is evidently observed. Its intensity conversion efficiency is 6.88×10^{-11}, corresponding to an effective nonlinear susceptibility $\chi_{eff}^{(2)}$ of 1.54×10^{3} pm V^{-1}, which exceeds the value of GaAs by over one order of magnitude and is comparable to that of $LaTiO_3$[25]. As a good insulator, sapphire possesses a much larger bandgap (≈8.0 eV) than silicon, meaning fewer electrons are initially present in the conduction band, and fewer new electrons are generated by impact ionization under an equivalent THz field. It leads to a nearly four three orders of magnitude weaker harmonic intensity than that of the undoped-silicon-based metamaterial. Despite this, the experimental results fully suggest that the proposed mechanism of second-order optical nonlinearity from the Thomson scattering is applicable to both semiconductors and dielectrics.

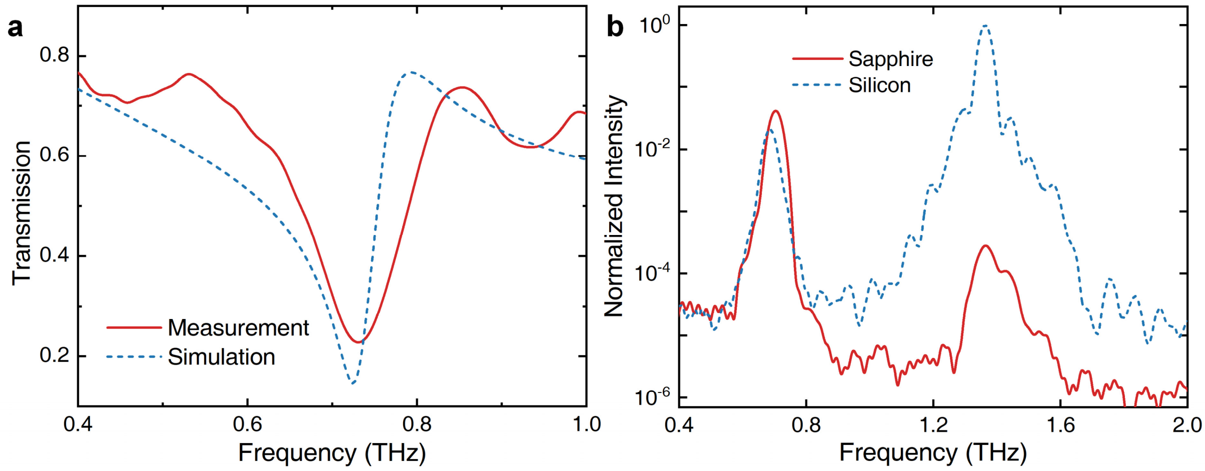

Supplementary Figure 9: Linear and nonlinear characterizations for the sapphire-based metamaterial sample. a, Simulated and measured linear transmission spectra for the sapphire-based sample; **b**, measured nonlinear spectra for the sapphire- and silicon-based samples, normalized to the SHG peak of the latter.

The much lower conversion efficiency of SHG experimentally rules out the possibility that the second-order nonlinear responses principally arise from the surface nonlinearity of gold SRRs as well. If it was dominated by the surface symmetry breaking of gold, one would expect comparable SHG strength independently from the substrate materials due to the same SRR structure, as previously reported in the near infrared and visible regimes[26-28]. The significant difference in terms of measured conversion efficiency between the substrates demonstrates the essence of our nonlinear responses is fundamentally different from previously proposed mechanisms.

Supplementary Note 12: SHG at different THz frequencies

We designed several samples fabricated on an undoped silicon substrate and operating at different frequencies to demonstrate the wide applicability of the nonlinear mechanism in the THz spectral range. We scaled up and down the geometric constant of the SRR structure to configure the resonance of the metamaterial at lower and higher frequencies, respectively, as shown in Supplementary Figs. 10a and c. The linear transmission spectra of the two new samples are plotted in Supplementary Figs. 10b and d, and the resonant frequencies at 0.47 THz and 0.96 THz for the larger and smaller structures respectively.

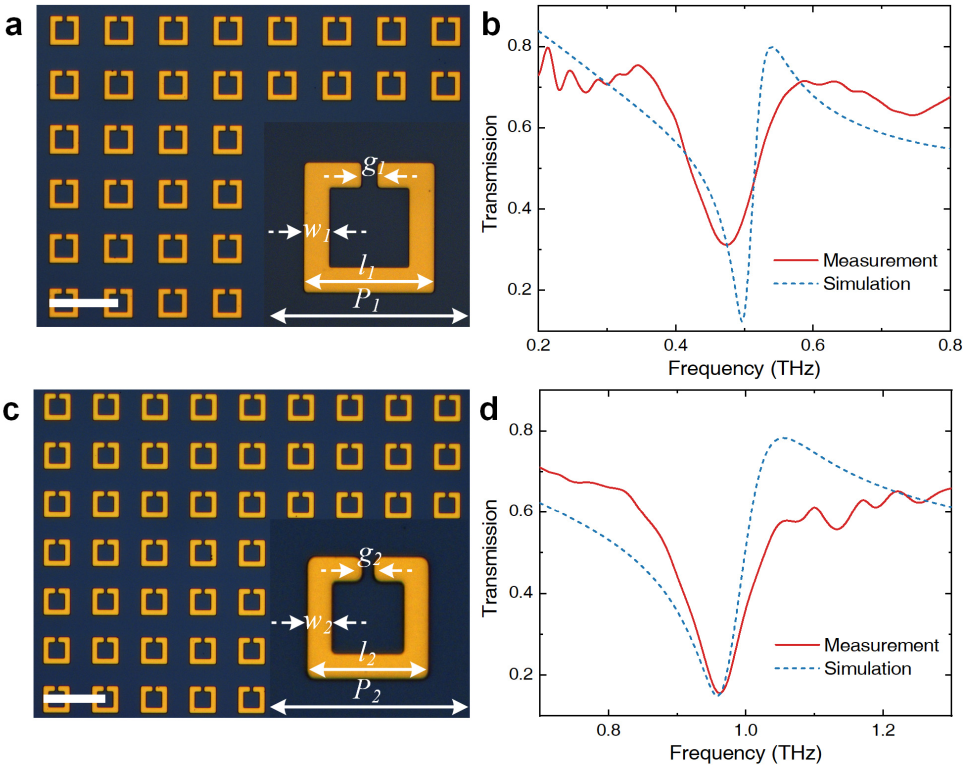

Supplementary Figure 10: Metamaterial samples resonating at different frequencies. a, Microscopic image of the sample resonating at 0.47 THz (P_1=80 μm, l_1=42 μm, w_1=7.5 μm, g_1=6 μm); **b**, its simulated and measured linear transmission spectra. **c**, Microscopic image of the sample resonating at 0.96 THz (P_2=40 μm, l_2=21 μm, w_2=3.5 μm, g_2=3 μm); **d**, its simulated and measured linear transmission spectra. Scale bars: **a**, 100 μm; **c**, 50 μm.

The nonlinear cross-polarized spectra of the two metamaterial samples were both measured with an intense THz illumination at 0.5 THz and 1.0 THz with the peak amplitude around 100 kV cm^{-1}, as revealed in Supplementary Figs. 11a and b (red curves), respectively. As expected, the SHG signals at 1.0 THz and 2.0 THz can be observed in the larger and smaller samples, respectively. These results prove that by judiciously modifying the metamaterial geometry, the second-order nonlinear responses can be configured to other frequencies benefitting from the wide applicability of the impact ionization and the nonlinear Thomson scattering mechanisms described above.

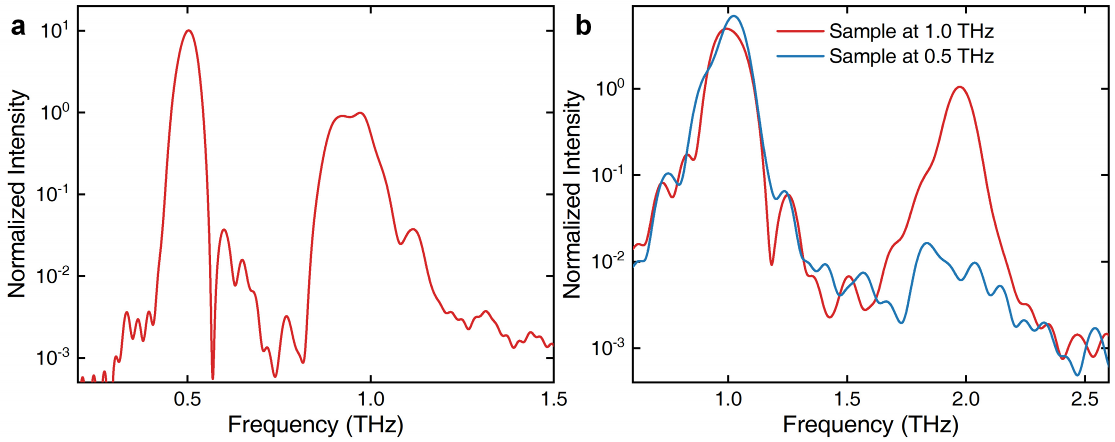

Supplementary Figure 11: Nonlinear responses for the metamaterial samples at different frequencies. a, Measured intensity spectrum of the larger sample pumped at a fundamental frequency of 0.5 THz, normalized to its peak; **b**, measured intensity spectra of both the smaller and the larger samples, pumped at the same fundamental frequency of 1.0 THz, and normalized to the peak recorded in the smaller sample.

We also pumped the sample working at 0.5 THz with a fundamental wave centered at 1.0 THz, the nonlinear spectrum of which is shown in Supplementary Fig. 11b (blue curve), and found that the SHG is barely observable. The sharp contrast in terms of SHG intensity between the on- and off-resonance illuminations clearly manifests the fundamental role of the metamaterial in tailoring the second-order nonlinear responses. Without the local enhancement of the magnetic and electric fields induced by the resonance, both impact ionization and Thomson scattering vanish.

Supplementary References

1 Hirori, H. *et al.* Extraordinary carrier multiplication gated by a picosecond electric field pulse. *Nat. Commun.* **2**, 594, (2011).

2 Vicario, C., Shalaby, M. & Hauri, C. P. Subcycle Extreme Nonlinearities in GaP Induced by an Ultrastrong Terahertz Field. *Phys. Rev. Lett.* **118**, 083901, (2017).

3 Anderson, C. L. & Crowell, C. R. Threshold Energies for Electron-Hole Pair Production by Impact Ionization in Semiconductors. *Phys. Rev. B* **5**, 2267-2272, (1972).

4 Zhao, H. *et al.* Ultrafast hydrogen bond dynamics of liquid water revealed by terahertz-induced transient birefringence. *Light Sci. Appl.* **9**, 136, (2020).

5 Shalaby, M. & Hauri, C. P. Demonstration of a low-frequency three-dimensional terahertz bullet with extreme brightness. *Nat. Commun.* **6**, 5976, (2015).

6 Okuto, Y. & Crowell, C. R. Energy-Conservation Considerations in the Characterization of Impact Ionization in Semiconductors. *Phys. Rev. B* **6**, 3076-3081, (1972).

7 Kang, B. J. *et al.* Ultrafast and Low-Threshold THz Mode Switching of Two-Dimensional Nonlinear Metamaterials. *Nano Lett.* **22**, 2016-2022, (2022).

8 Ganichev, S. *et al.* Impact ionization in semiconductors under the influence of the electric field of an optical wave. *Soviet Physics JETP* **63**, 256-263, (1986).

9 Ershov, M. & Ryzhii, V. Temperature dependence of the electron impact ionization coefficient in silicon. *Semicond. Sci. Tech.* **10**, 138-142, (1995).

10 Enke, L., Bingsheng, Z. & Jinsheng, L. Physics of semiconductor. *Publishing House of Electronics Industry* **133**, (2003).

11 Wolfe, J. P. Thermodynamics of excitons in semiconductors. *Phys. Today* **35**, 46-54, (1982).
12 Maes, W., De Meyer, K. & Van Overstraeten, R. Impact ionization in silicon: A review and update. *Solid State Electron.* **33**, 705-718, (1990).
13 Boyd, R. W. *Nonlinear optics*. (Academic press, 2008).
14 He, G. S. & Liu, S. H. *Physics of nonlinear optics*. Vol. 216 (World Scientific, 1999).
15 Yan, W. *et al.* High-order multiphoton Thomson scattering. *Nat. Photonics* **11**, 514-520, (2017).
16 Babzien, M. *et al.* Observation of the second harmonic in Thomson scattering from relativistic electrons. *Phys. Rev. Lett.* **96**, 054802, (2006).
17 Lau, Y., He, F., Umstadter, D. P. & Kowalczyk, R. Nonlinear Thomson scattering: A tutorial. *Phys. Plasmas.* **10**, 2155-2162, (2003).
18 Chen, S.-y., Maksimchuk, A. & Umstadter, D. Experimental observation of relativistic nonlinear Thomson scattering. *Nature* **396**, 653, (1998).
19 Li, G., Zhang, S. & Zentgraf, T. Nonlinear photonic metasurfaces. *Nat. Rev. Mater.* **2**, 17010, (2017).
20 Ciracì, C., Poutrina, E., Scalora, M. & Smith, D. R. Origin of second-harmonic generation enhancement in optical split-ring resonators. *Phys. Rev. B* **85**, 201403, (2012).
21 Ciracì, C., Poutrina, E., Scalora, M. & Smith, D. R. Second-harmonic generation in metallic nanoparticles: Clarification of the role of the surface. *Phys. Rev. B* **86**, (2012).
22 Berciaud, S., Cognet, L., Tamarat, P. & Lounis, B. Observation of Intrinsic Size Effects in the Optical Response of Individual Gold Nanoparticles. *Nano Lett.* **5**, 515-518, (2005).
23 Guyot-Sionnest, P., Chen, W. & Shen, Y. R. General considerations on optical second-harmonic generation from surfaces and interfaces. *Phys. Rev. B* **33**, 8254-8263, (1986).
24 Klaassen, D. A unified mobility model for device simulation—II. Temperature dependence of carrier mobility and lifetime. *Solid State Electron.* **35**, 961-967, (1992).
25 Mayer, A. & Keilmann, F. Far-infrared nonlinear optics. I. chi (2) near ionic resonance. *Phys. Rev. B* **33**, 6954-6961, (1986).
26 Klein, M. W., Enkrich, C., Wegener, M. & Linden, S. Second-harmonic generation from magnetic metamaterials. *Science* **313**, 502-504, (2006).
27 Krasnok, A., Tymchenko, M. & Alù, A. Nonlinear metasurfaces: a paradigm shift in nonlinear optics. *Materials Today* **21**, 8-21, (2018).
28 Lapine, M., Shadrivov, I. V. & Kivshar, Y. S. Colloquium: Nonlinear metamaterials. *Rev. Mod. Phys.* **86**, 1093-1123, (2014).
